# ZEB1 protects skeletal muscle from damage and is required for its regeneration

**DOI:** 10.1038/s41467-019-08983-8

**Published:** 2019-03-25

**Authors:** Laura Siles, Chiara Ninfali, Marlies Cortés, Douglas S. Darling, Antonio Postigo

**Affiliations:** 1 Group of Transcriptional Regulation of Gene Expression, Department of Oncology and Hematology, IDIBAPS, 08036 Barcelona, Spain; 2 Center for Genetics and Molecular Medicine and Department of Immunology and Infectious Diseases, University of Louisville, Louisville, KY 40202 USA; 3 Molecular Targets Program, James G. Brown Cancer Center, Louisville, KY 40202 USA; 4 ICREA, Barcelona, 08010 Spain

## Abstract

The mechanisms linking muscle injury and regeneration are not fully understood. Here we report an unexpected role for ZEB1 regulating inflammatory and repair responses in dystrophic and acutely injured muscles. ZEB1 is upregulated in the undamaged and regenerating myofibers of injured muscles. Compared to wild-type counterparts, *Zeb1*-deficient injured muscles exhibit enhanced damage that corresponds with a retarded p38-MAPK-dependent transition of their macrophages towards an anti-inflammatory phenotype. *Zeb1*-deficient injured muscles also display a delayed and poorer regeneration that is accounted by the retarded anti-inflammatory macrophage transition and their intrinsically deficient muscle satellite cells (MuSCs). Macrophages in *Zeb1*-deficient injured muscles show lower phosphorylation of p38 and its forced activation reverts the enhanced muscle damage and poorer regeneration. MuSCs require ZEB1 to maintain their quiescence, prevent their premature activation following injury, and drive efficient regeneration in dystrophic muscles. These data indicate that ZEB1 protects muscle from damage and is required for its regeneration.

## Abstract (web summary)

Following muscle damage, an inflammatory response is associated to activation of satellite cells, which drive muscle repair. Here, the authors show that upregulation of Zeb1 in macrophages and muscle fibres regulates inflammation, and also show a role for Zeb1 in maintenance of satellite cell quiescence.

## Introduction

The capacity of skeletal muscle to regenerate in response to damage lies on its progenitor cells known as muscle satellite cells (MuSCs)^1,2^. MuSCs are normally quiescent but they become activated, proliferate, and differentiate in response to stress and injury^1–3^. In muscle dystrophies, myofibers display greater fragility and undergo continuous cycles of degeneration, inflammation, and progressively impaired regeneration^4^. In addition to structurally unstable myofibers, deficient regeneration in patients with Duchenne muscular dystrophy (DMD), and in the mdx mouse, an experimental model of DMD, is also related to functionally defective MuSCs^3,5^.

During muscle damage the release of soluble factors—notably, the CCL2 chemokine—by injured myofibers, as well as by activated MuSCs and stromal cells prompts the recruitment and infiltration of circulating immune cells, mainly monocytes^6–13^. The inflammatory milieu at the site of injury (e.g., Tumor Necrosis Factor-α [TNFα], Interferon-γ [IFNγ]) both activates monocytes toward pro-inflammatory macrophages (F4/80^+^, Ly6C^high^) and triggers the expansion of MuSCs—also referred at that point as proliferating myoblasts—while blocking their differentiation^14,15^. The subsequent decline of TNFα and IFNγ levels and the increase of IL10 promote the transition of pro-inflammatory macrophages toward an anti-inflammatory phenotype (F4/80^+^, Ly6C^low^, MRC1/CD206^+^), a requisite for MuSCs to begin their differentiation^7,16–18^. This macrophage switch is driven by the activation of the p38-MAPK and its balance with DUSP1 (MKP-1)^17^. In cycling myoblasts, MyoD activates proliferation-associated genes but not differentiation genes, whose regulatory regions are repressed by ZEB1 (also known as δEF1 and ZEB) and SNAI1/SNAI2 transcription factors^19–21^. Only after myoblasts have exited the cell cycle, MyoD displaces ZEB1 and SNAI1/SNAI2 from these genes to drive myoblast differentiation into myofibers.

ZEB1 is best known for triggering an epithelial-to-mesenchymal transition (EMT) in cancer cells to promote tumor progression^22,23^. ZEB1 also plays important roles in embryogenesis—*Zeb1* (−/−) mice die before birth—and it is expressed in the primary myotome, where the first muscle progenitors arise^24^. ZEB1 imposes a stage-dependent inhibition of muscle differentiation, so *Zeb1* (−/−) embryos exhibit the premature expression of adult muscle differentiation genes^21^. Interestingly, ZEB1 maintains stemness in cancer cells^25,26^. However, the expression and role of ZEB1 in the specification and differentiation of normal adult stem cells, including MuSCs, or its potential role in tissue regeneration have not been explored.

The above evidence prompted us to question whether ZEB1 plays a role in MuSC myogenic progression in the context of muscle injury and regeneration. Using a chronic muscular dystrophic mouse [mdx (*Dmd*^*mdx*^)] and a model of acute muscle injury, we show that ZEB1 protects skeletal muscle from damage and is required for its regeneration. ZEB1 is upregulated in injured muscles being expressed by undamaged and regenerating myofibers. Downregulation of *Zeb1* in mice [*Zeb1* (+/−)] results in an increased and more prolonged immune infiltration and damage of their muscles in response to injury, as well as in a retarded and poorer muscle regeneration. ZEB1 transcriptionally represses the *Ccl2* promoter and, compared to wild-type counterparts, *Zeb1* (+/−) injured muscles show increased CCL2 secretion by their myofibers and MuSCs. Infiltrating macrophages from *Zeb1* (+/−) injured muscles display a retarded transition to an anti-inflammatory phenotype, which corresponded to a deficient upregulation of phosphorylated p38-MAPK and of *Dusp1* in response to injury. In vivo forced activation of p38 in *Zeb1* (+/−) injured muscles revert their enhanced damage and poorer regeneration to the same levels than in wild-type injured muscles. Delayed and poorer regeneration in *Zeb1* (+/−) injured muscles is accounted by the retarded transition of *Zeb1* (+/−) macrophages, as well as their functional deficient MuSCs. MuSCs require ZEB1 to maintain their quiescence—via the inhibition of *Myod1*, the transcriptional activation of *Foxo3*, and the upregulation of Notch target genes—and for the efficient engraftment and regeneration of damaged muscles.

Therapeutic approaches to muscular dystrophies aim both to modulate the inflammatory response and to improve MuSCs’ regenerative capacity. However, the mechanisms linking both processes are still not fully understood. Our results reveal an unexpected role for ZEB1 regulating the inflammatory and repair responses during muscle damage and can potentially open new strategies in the treatment of muscular dystrophies.

## Results

### ZEB1 is upregulated in dystrophic muscles and is expressed by undamaged myofibers

**Fig. 1:**
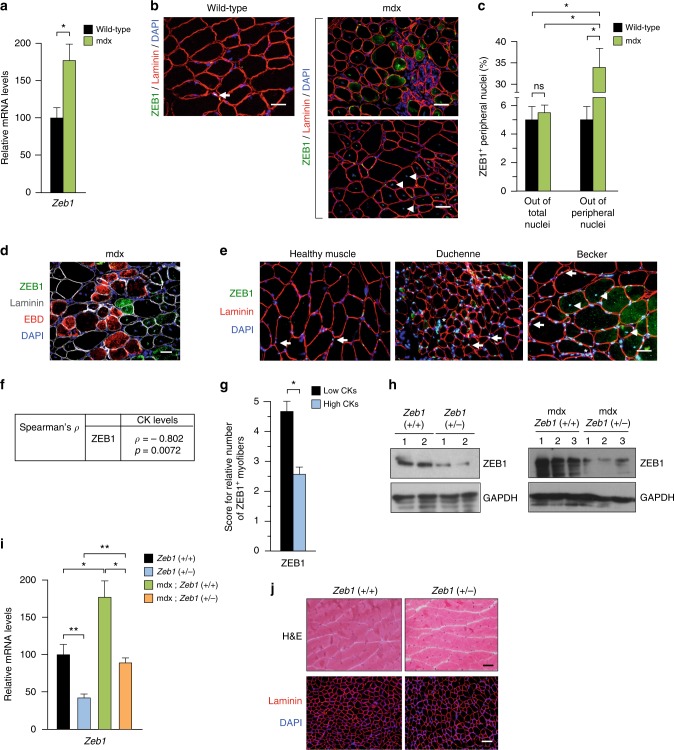
ZEB1 is upregulated in dystrophic muscles and is expressed by undamaged myofibers. **a** *Zeb1* mRNA levels in the gastrocnemius muscles of 2-month-old wild-type and mdx mice were assessed by qRT-PCR. Data are the average of six mice for each genotype. Throughout the Figures, relative data in percentage are shown with the value of the wild-type or control condition arbitrarily set to 100. **b** The gastrocnemius muscles of wild-type and mdx mice were assessed for ZEB1 (clone H102) and laminin (4H8-2) along with DAPI for nuclear staining. Representative peripheral and centralized nuclei were labeled with arrows and arrowheads, respectively. For mdx muscles, two different areas are shown: one that predominantly exhibits damaged fibers (upper panel), and another with signs of regeneration (lower panel). See Supplementary Fig. 1A for individual staining captures. Scale bars: 25 μm (wild-type mice) 50 μm (mdx mice). **c** The percentage of ZEB1^+^ peripheral nuclei in **b** was calculated either out of the total number of nuclei (peripheral plus centralized) or only with respect to peripheral nuclei. Data are the mean of at least five fields from three mice for each genotype. **d** As in **b**, but 9–12 h before euthanasia mice were injected with EBD. See Supplementary Fig. 1C for individual staining. Scale bar: 50 μm. **e** Human healthy and dystrophic muscles were stained for ZEB1 (HPA027524) and laminin (4H8-2) along with DAPI. Representative peripheral and centralized nuclei were labeled with arrows and arrowheads, respectively. A representative area with immune cell infiltration is labeled with an asterisk (“*”). See Supplementary Fig. 1D for single staining captures. Scale bar: 50 μm. **f** Correlation between ZEB1 expression and CK levels in dystrophic human muscles. **g** Relative number of fibers expressing ZEB1 in human dystrophic muscles with respect to their CK levels below or above the median. See Supplementary Fig. 1E for representative scores of ZEB1 staining. **h** *Left panel:* Gastrocnemius muscle lysates from 2-month-old wild-type and *Zeb1* (+/−) mice (two per genotype, labeled as 1 and 2) were blotted for ZEB1 (HPA027524) and GAPDH (14C10) as loading control. *Right panel*: As in the left panel but from 2-month-old mdx;*Zeb1* (+/+) and mdx;*Zeb1* (+/−) mice, three for each genotype. See Supplementary Fig. 1F for full unedited blots. **i** *Zeb1* mRNA levels in the gastrocnemius of the four genotypes were determined by qRT-PCR. Data are the average of six mice per genotype. **j** Wild-type and *Zeb1* (+/−) gastrocnemius muscles were either counterstained with hematoxylin/eosin (H&E) (*upper panel*) or immunostained for laminin (4H8-2) and DAPI (*lower panel*). Scale bars: 50 and 100 μm, respectively

We first examined ZEB1 expression in the gastrocnemius of wild-type and mdx mice and found that *Zeb1* messenger RNA (mRNA) was upregulated in dystrophic muscles (Fig. 1a). In the healthy muscle of wild-type mice, ZEB1 was restricted to a subset of peripheral nuclei (a representative nucleus is labeled with an arrow in Fig. 1b and in Supplementary Fig. 1a). In contrast, in areas of mdx muscles with morphological signs of damage, ZEB1 was expressed not only in peripheral nuclei but also in the cytoplasm of some fibers (Fig. 1b and Supplementary Fig. 1a). Notably, ZEB1 was not expressed in mdx damaged myofibers and/or with infiltration by immune cells.

In contrast to human dystrophic muscles, where muscle repair is more limited, muscles in young mdx mice display numerous centralized nuclei, a marker of regeneration^27^. Many of these centralized nuclei were also positive for ZEB1 (arrowheads in Fig. 1b and in Supplementary Fig. 1A, B). The percentage of ZEB1^+^ peripheral nuclei was higher in mdx muscles than in wild-type ones (Fig. 1c). Of note, a given myofiber can harbor both ZEB1^+^ and ZEB1^–^ nuclei (Supplementary Fig. 1b). ZEB1 was also examined in mdx mice that before euthanasia had been injected with Evans blue (EBD), a red fluorescent dye that is incorporated in vivo by damaged myofibers. ZEB1 was expressed by undamaged myofibers but not by EBD^+^ damaged fibers (Fig. 1d and Supplementary Fig. 1c). To summarize, ZEB1 was upregulated in dystrophic muscles (Fig. 1a), but its expression was restricted to undamaged or regenerating myofibers (Fig. 1b, d).

As in wild-type mice, ZEB1 was found in a subset of peripheral nuclei of healthy human muscles (arrows in Fig. 1e and Supplementary Fig. 1d). In turn, and in parallel with the mdx mouse, in human muscular dystrophies, ZEB1 was expressed not only in peripheral nuclei but also in centralized nuclei and in the cytoplasm of some fibers (Fig. 1e and Supplementary Fig. 1d).

Tissue damage in muscle dystrophies is accompanied by elevated serum levels of creatine kinase (CK)^4^. Interestingly, CK levels in muscular dystrophy patients maintained a strong negative correlation with ZEB1 (Spearman’s *ρ*: −0.80) (Fig. 1f). The number of ZEB1^+^ myofibers was higher among patients with lower CKs (Fig. 1g and Supplementary Fig. 1e). These data suggest that ZEB1 expression in human and mouse dystrophic muscles associates with reduced damage.

### ZEB1 protects dystrophic muscles from damage

ZEB1’s tumor promoting functions depend on a fine threshold of its expression and the deletion of one *Zeb1* allele in either cancer cells or tumor-associated macrophages is sufficient to block tumor progression in *Zeb1* (+/−) mice^28–30^. Here, we also used the *Zeb1* (+/−) mouse model to investigate if the role of ZEB1 in normal and injured muscle depends on a similarly fine threshold. Gastroc-nemius muscles in *Zeb1* (+/−) mice—that expresses around half of ZEB1 levels than in wild-type mice (Fig. 1h, i and *left panel* of Supplementary Fig. 1f)—displayed normal weight, and normal macroscopic and histological structure (Fig. 1j and Supplementary Fig. 1g, h). Nevertheless, *Zeb1* (+/−) myofibers have a larger average size than wild-type counterparts with fewer smaller size fibers and more larger size ones (Supplementary Fig. 1i, j).

To test whether ZEB1 protects dystrophic muscle from damage, *Zeb1* expression was downregulated in mdx mice by crossing them with *Zeb1* (+/−) ones to generate mdx;*Zeb1* (+/−) mice (Fig. 1h, i and *right panel* of Supplementary Fig. 1f). Compared to wild-type counterparts, mdx mice younger than 2.5-months have lighter muscles but in older mdx mice muscles are heavier and their myofibers larger^31,32^. The gastrocnemius muscles of 2-months-old mdx;*Zeb1* (+/−) mice were lighter and have a higher proportion of smaller size myofibers than those in mdx mice with full levels of *Zeb1* [hereafter referred as mdx;*Zeb1* (+/+)] (Supplementary Fig. 2a, b). However, at 10–15 months of age, mdx;*Zeb1* (+/−) muscles were heavier and have a higher proportion of larger size myofibers than mdx;*Zeb1* (+/+) counterparts (Supplementary Fig. 2a, c, d).

**Fig. 2:**
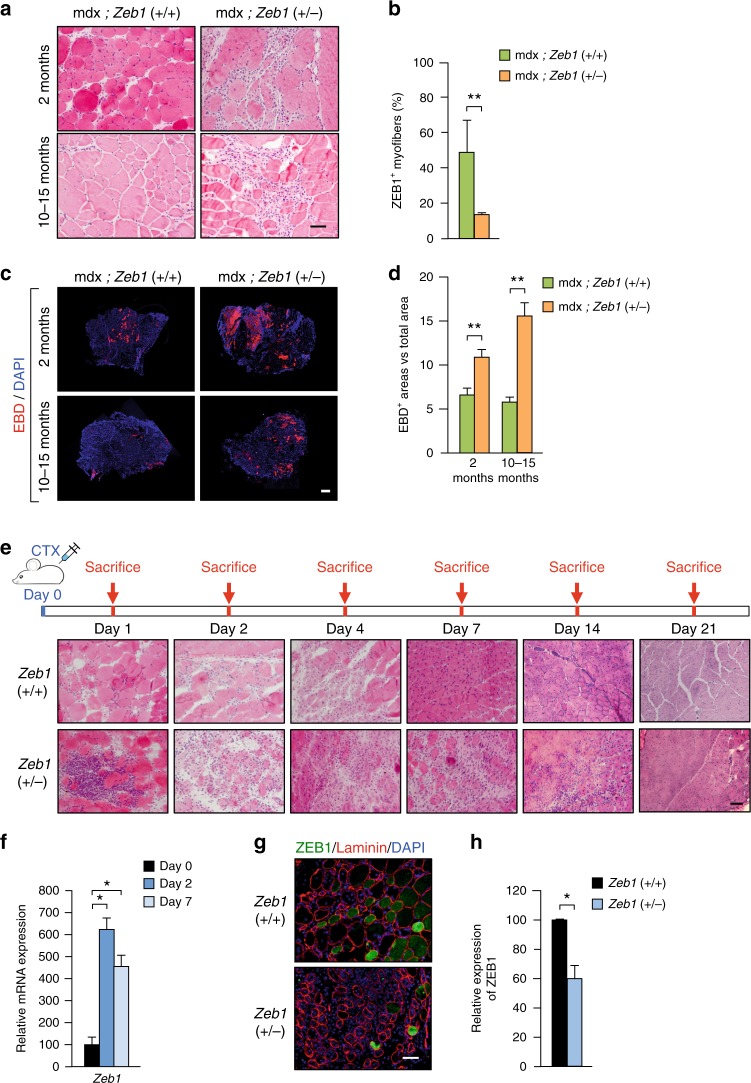
ZEB1 protects dystrophic and acutely injured muscles from damage. **a** The gastrocnemius muscles from 2-month- and 10–15-month-old mdx;*Zeb1* (+/+) and mdx;*Zeb1* (+/−) mice were stained for hematoxylin/eosin. Representative pictures from at least ten mice for each genotype. Scale bar: 50 μm. **b** ZEB1 expression (H102) in 2-month-old mdx;*Zeb1* (+/+) and mdx;*Zeb1* (+/−) gastrocnemius was assessed by immunofluorescence as in Supplementary Fig. 2F. **c** As in **a**, but 9–12 h before euthanasia mice were injected with EBD. Samples were also stained for DAPI. Representative figures from at least eight mice per genotype. Scale bar: 400 μm. **d** Quantification of EBD^+^ areas in **c**. **e** The gastrocnemius of 2-month-old wild-type and *Zeb1* (+/−) mice were injected with CTX. Mice were then euthanized at the indicated times to assess muscular histological alterations by hematoxylin/eosin staining. Representative captures for at least three mice per genotype and day. Scale bar: 50 μm. **f** Two-month-old wild-type mice were injected with CTX and *Zeb1* mRNA levels were assessed by qRT-PCR in at least four mice per genotype. **g** The gastrocnemius muscles of wild-type and *Zeb1* (+/−) mice were assessed for ZEB1 (H102) and laminin (4H8-2) expression along with DAPI. Representative merged pictures of four mice per genotype. See Supplementary Fig. 2G for single staining. Scale bar: 50 μm. **h** Quantification of ZEB1^+^ myofibers per field in **g**

Dystrophic muscles display histologic abnormalities and greater fiber size variability than healthy muscles^4^. Accordingly, and compared to wild-type ones (Fig. 1j), the gastrocnemius of mdx mice—independently of *Zeb1* levels—exhibited myofibers of widely different sizes and areas of degeneration, inflammation, and regeneration (Fig. 2a and Supplementary Fig. 2e). However, fiber damage and inflammatory infiltration was more intense and extensive in mdx;*Zeb1* (+/−) muscles than in mdx;*Zeb1* (+/+) ones (Fig. 2a). As expected, expression of ZEB1 was lower in mdx;*Zeb1* (+/−) muscles (Fig. 2b and Supplementary Fig. 2f). EBD staining confirmed that the damaged area in mdx;*Zeb1* (+/−) muscles was larger than in mdx;*Zeb1* (+/+) muscles (Fig. 2c, d). A decline over time in the EBD staining of mdx muscles has been reported (e.g., ref. ^33^). Altogether, these data indicate that ZEB1 has a protective role in dystrophic mdx muscles while its downregulation in the mdx;*Zeb1* (+/−) mouse enhanced myofiber damage.

### ZEB1 protects muscle against acute exogenous injury

We then evaluated the response of wild-type and *Zeb1* (+/−) muscles to the injection of the snake venom cardiotoxin (CTX), a well-established model of acute muscle injury^34,35^. During the first 2 days, wild-type and *Zeb1* (+/−) muscles displayed local necrosis and abundant inflammatory infiltrate but both processes were more intense and extensive in *Zeb1* (+/−) muscles (Fig. 1j for day 0 and Fig. 2e). By day 4 post-injection, muscles started to show signs of regeneration but necrosis and inflammation remained higher in *Zeb1* (+/−) mice. By day 7, wild-type muscles had most of their inflammatory infiltration already resolved and numerous myofibers had centralized nuclei, indicative of regeneration. In contrast, *Zeb1* (+/−) muscles still showed areas of necrosis and inflammatory infiltrate, as well as abnormal regeneration with myofibers of diverse sizes. Fourteen days after CTX injection, the overall histological architecture of wild-type muscles have been largely restored; meantime, *Zeb1* (+/−) muscles exhibited scattered regions of necrosis and infiltrate along with regenerating areas. Despite enhanced damage in *Zeb1* (+/−) muscles, their myofibers were smaller and fewer of them displayed centralized nuclei suggesting a compromised and/or delayed muscle regeneration (see below). Lastly, 21 days after injury, muscles from both genotypes achieved nearly complete regeneration although scattered centralized nuclei are still visible, particularly in *Zeb1* (+/−) muscles.

As in dystrophic mdx muscles (Fig. 1a), *Zeb1* mRNA increased following CTX injection (Fig. 2f). Likewise, *Zeb1* (+/−) injured muscles contained fewer ZEB1^+^ myofibers than wild-type injured muscles (Fig. 2g, h and Supplementary Fig. 2g). Again, ZEB1 expression associated with reduced muscle damage. Like in dystrophic muscles (Fig. 1b, d, e), ZEB1 was expressed in non-infiltrated fibers of CTX-injured muscles but not in damaged fibers (Fig. 2g and Supplementary Fig. 2g). Higher inflammatory infiltration in *Zeb1* (+/−) gastrocnemius translated into heavier muscles following CTX-induced injury (Supplementary Fig. 2h). Altogether, these results suggest that—in both dystrophic and acutely injured muscles—ZEB1 inhibits immune cell infiltration, reduces muscle damage, and accelerates the resolution of inflammation.

### IGF-1 upregulates ZEB1 and promotes its ERK/MEK-dependent cytoplasmic translocation

Despite being a transcription factor, ZEB1 can also be found in the cytoplasm of some cancer cells, probably reflecting that high levels of ZEB1 saturate the nuclear translocation system^36–38^. However, the potential role of ZEB1 (if any) in the cytoplasm of cancer cells remains unknown.

**Fig. 3:**
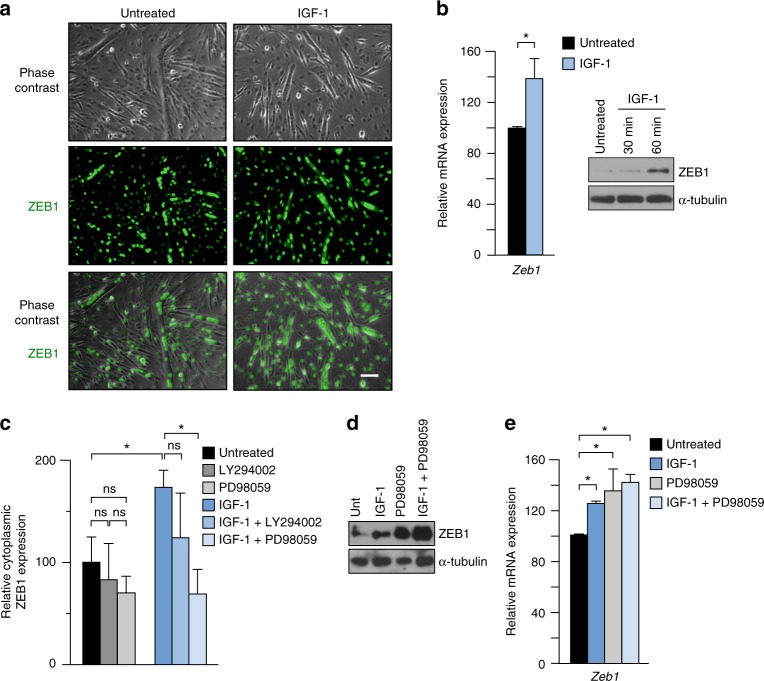
IGF-1 upregulates ZEB1 and promotes its ERK/MEK-dependent cytoplasmic translocation. **a** C2C12 myotubes were cultured in the presence or absence of 100 ng/ml of IGF-1 for 1 h and the expression of ZEB1 (H-102) was assessed by immunofluorescence. See Supplementary Fig. 3A for DAPI staining and additional staining combinations. Representative captures of four independent experiments. Scale bar: 50 μm. **b** *Left panel*: *Zeb1* mRNA levels were determined by qRT-PCR in C2C12 myotubes cultured in the presence or absence of 100 ng/ml of IGF-1 for 1 h. *Right panel*: ZEB1 protein (HPA027524) levels in C2C12 myotubes cultured in the absence of presence of IGF-1 for 30 or 60 min were assessed by western blot along with α-tubulin (T6074). Representative blots from four independent experiments. See Supplementary Fig. 3B for the full unedited blot. **c** As in **a**, C2C12 myotubes were incubated during 1 h with or without 100 ng/ml of IGF-1 and/or either 20 μm of LY294002 or 40 μm of PD98059. Cells were assessed for their ZEB1 cytoplasmic expression by immunofluorescence as in **a**. Data shown are the average of five fields per condition from four independent experiments. **d** As in **c**, C2C12 myotubes were treated with or without IGF-1 and/or PD98059 and cell lysates were blotted for ZEB and α-tubulin as in **b**. Blots are the representative of four independent experiments. Supplementary Fig. 3C for full unedited blots. **e** As in **d**, but *Zeb1* expression was determined by qRT-PCR. Data are the average of four independent experiments

The above data indicated that some non-infiltrated myofibers of injured muscles also expressed ZEB1 in their cytoplasm. In some fibroblastic and epithelial established cell lines, the cellular localization of selective truncated ZEB1 peptides is regulated through phosphorylation by phorbol ester and IGF-1^38^. Interestingly, IGF-1 downregulates the inflammatory response following muscle injury and accelerates muscle regeneration^39^. It was found here that IGF-1 increased ZEB1 expression in C2C12 myotubes and promoted its partial translocation to the cytoplasm (Fig. 3a, b and Supplementary Fig. 3a, b). Given that IGF-1 signals through PI3K and MEK/ERK pathways, C2C12 myotubes were incubated in the presence or absence of IGF-1 and/or inhibitors of PI3K (LY294002) or of MEK/ERK (PD98059). The partial cytoplasmic translocation of ZEB1 induced by IGF-1 was inhibited by PD98059—which also upregulated ZEB1 expression—but not by LY294002 (Fig. 3c–e and Supplementary Fig. 3c). These data indicate that IGF-1, through MEK/ERK, upregulates ZEB1 and increases its cytoplasmic localization.

### ZEB1 inhibits the expression of CCL2 and pro-inflammatory markers in response to injury

**Fig. 4:**
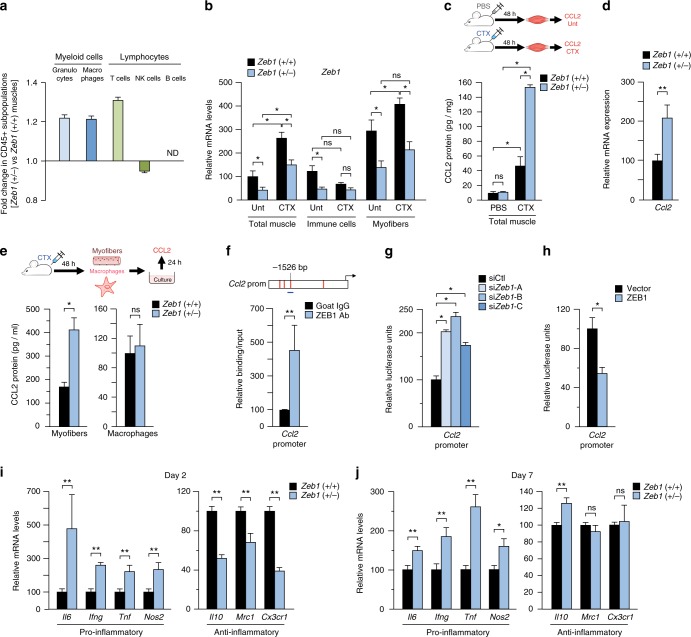
ZEB1 inhibits the expression of CCL2 and pro-inflammatory markers in response to injury. **a** Analysis by FACS of immune cell subpopulations in 2-month-old wild-type and *Zeb1* (+/−) gastrocnemius 48 h after CTX injection. Histograms represent the fold change of each subpopulation in *Zeb1* (+/−) muscles with respect to wild-type counterparts. Data originated from three mice per genotype. **b** Ze*b1* mRNA levels in total muscle, in isolated immune (CD45^+^) cells, and in isolated myofibers from untreated and CTX-injured wild-type and *Zeb1* (+/−) muscles after 48 h. Data are the average of at least three mice per genotype and condition. **c** Wild-type and *Zeb1* (+/−) mice were injected with PBS or CTX and 48 h later their gastrocnemius were assessed for CCL2 production by ELISA. Data are the mean of at least three mice per genotype and condition. **d** As in **b**, wild-type and *Zeb1* (+/−) gastrocnemius were assessed for *Ccl2* mRNA levels by qRT-PCR 48 h after CTX injection. Data are the mean of at least five mice per genotype. **e** Wild-type and *Zeb1* (+/−) mice were injected with CTX and 48 h later their myofibers and macrophages were isolated and their secretion of CCL2 after 24 h of ex vivo culture was quantified by ELISA. Data are the mean of four mice per genotype. **f** *Upper panel*: Scheme of 2 kb of the mouse *Ccl2* promoter. Consensus binding sites for ZEB1 are marked as vertical red lines. The promoter region assessed by ChIP for a ZEB1 binding site at −1526 bp (Supplementary Methods) is represented by a horizontal blue line. *Lower panel*: DNA from C2C12 cells was immunoprecipitated with antibodies against ZEB1 (E-20) or control goat IgG and amplified by qRT-PCR for the indicated *Ccl2* promoter region. Data are the mean of five independent experiments. **g** C2C12 myoblasts were transiently interfered with 50–100 nM of a siRNA control (siCtl) or siRNAs against *Zeb1* (si*Zeb1*-A, si*Zeb1*-B, si*Zeb1*-C) and co-transfected with 0.4 μg of a luciferase reporter for the mouse *Ccl2* promoter. ZEB1 knockdown is shown in Supplementary Fig. 4A. Data are representative of four independent experiments. **h** As in **g**, but C2C12 cells were transfected with 0.6 μg of an empty expression vector or the corresponding equal molar amount of the same vector encoding full-length *Zeb1*. Data are representative of four independent experiments. **i**, **j** Two or 7 days after CTX injection wild-type and *Zeb1* (+/−) gastrocnemius were assessed for the expression of the indicated genes by qRT-PCR. Data are the mean of at least five mice per genotype

During the first 24 h following an acute muscle injury, the inflammatory infiltrate is constituted mainly by granulocytes, which are later replaced by macrophages^7,13,40^. Figure 2a, e indicated that *Zeb1* downregulation increases the inflammatory infiltrate and delays its resolution. ZEB1 plays important roles in malignant lymphocytes and in tumor-associated macrophages^30,41,42^, but its expression and role in immune cells in the context of tissue damage and repair has not been studied. Even though *Zeb1* (+/−) CTX-injured muscles displayed, in absolute terms, higher inflammatory infiltration than wild-type muscles (Fig. 2e), FACS analysis did not find major differences in the relative distribution of the immune cell subpopulations (Fig. 4a). CTX injection upregulated *Zeb1* mRNA levels in isolated myofibers but not in isolated immune cells (Fig. 4b).

Secretion of the CCL2 chemokine, mainly by damaged myofibers but also by macrophages and activated MuSCs, drives the recruitment of circulating pro-inflammatory monocytes into acutely injured and dystrophic muscles^7,9,10,12,13^. Although CTX-induced injury upregulated CCL2 production in muscles of both genotypes, CCL2 levels were higher in *Zeb1* (+/−) muscles (Fig. 4c, d). The conditioned medium (CM) collected from *Zeb1* (+/−) myofibers—isolated from *Zeb1* (+/−) injured muscles and cultured for 24 h—contained more CCL2 than that obtained from wild-type myofiber cultures (Fig. 4e). In contrast, macrophages isolated from injured muscles of both genotypes secreted similar levels of CCL2 (Fig. 4e).

ZEB1 can either directly activate or repress the transcription of its target genes by recruitment of other transcription factors or non-DNA binding cofactors in a promoter- and tissue-specific manner^43–47^. We identified high-affinity ZEB1 binding sites in the first 2 kb fragment of the *Ccl2* promoter and confirmed the binding of ZEB1 to this promoter in chromatin immunoprecipitation (ChIP) assays (Fig. 4f). When C2C12 cells were transfected with a reporter containing the mouse *Ccl2* promoter fused to luciferase, knockdown of endogenous *Zeb1* with small interfering RNA (siRNA) (Supplementary Fig. 4a), upregulated *Ccl2* promoter’s activity, whereas *Zeb1* overexpression repressed it (Fig. 4g, h, respectively). Repression of *Ccl2* by ZEB1 is consistent with the higher inflammatory infiltration in *Zeb1* (+/−) injured muscles.

At day 2 post-CTX injection, *Zeb1* (+/−) muscles expressed higher levels of pro-inflammatory markers (*Il6, Ifng, Tnf, Nos2/INOS)* and lower of anti-inflammatory ones (*Il10, Mrc1/Cd206, Cx3cr1*) than wild-type muscles (Fig. 4i). Notably, 7 days after CTX injection, *Zeb1* (+/−) muscles still exhibited higher expression of pro-inflammatory markers (Fig. 4j), suggesting a delay in the switch of *Zeb1* (+/−) macrophages toward an anti-inflammatory phenotype.

### ZEB1 accelerates the switch of macrophages towards an anti-inflammatory phenotype through activation of p38

**Fig. 5:**
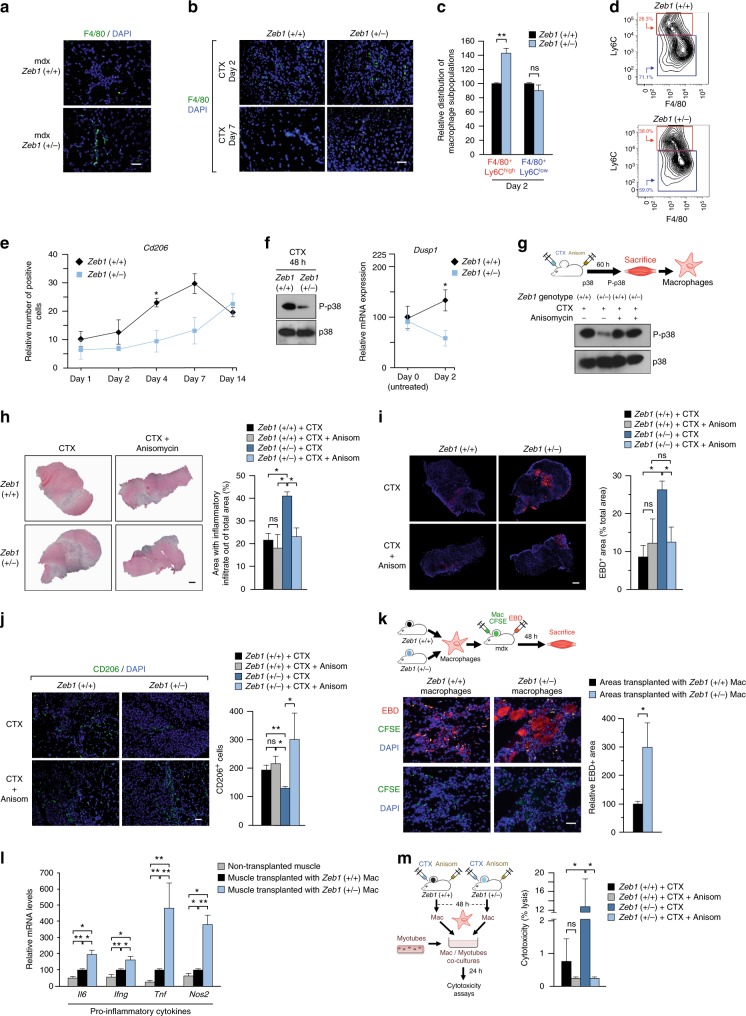
ZEB1 accelerates the p38-dependent transition of macrophages toward an anti-inflammatory phenotype and reduces their cytotoxic effect. **a** The gastrocnemius of mice from both genotypes were stained for F4/80 (BM8) along with DAPI. Scale bar: 50 μm. **b** As in **a**, but in wild-type and *Zeb1* (+/−) muscles 2 or 7 days after CTX injection. Scale bar: 50 μm. **c** The gastrocnemius of wild-type and *Zeb1* (+/−) mice were injected with CTX and infiltrating macrophages (CD11b^+^ F4/80^+^) were characterized for Ly6C (HK1.4) by FACS. Data are the mean of at least six mice per genotype. **d** Representative FACS plot for **c**. See Supplementary Fig. S5A for plots of other subpopulations. **e** Wild-type and *Zeb1* (+/−) gastrocnemius were assessed for CD206/MRC1 (MR5D3) expression up to 14 days following CTX injection (see Supplementary Fig. 5D). **f** *Left panel*: Lysates from macrophages isolated from wild-type and *Zeb1* (+/−) muscles 48 h after CTX injection were blotted for phosphorylated p38 (P-p38) (9211L) and total p38 (M0800). See Supplementary Fig. 5E for full unedited blots. Representative blots from three independent experiments. *Right panel*: *Dusp1* mRNA levels in wild-type and *Zeb1* (+/−) muscles before (day 0, untreated) and 2 days after CTX injection were determined by qRT-PCR. Data are the mean of at least four mice per genotype. **g** The gastrocnemius of wild-type and *Zeb1* (+/−) mice were injected 10 μm of CTX along with 15 μg of anisomycin per gram of body weight. Sixty hours later the infiltrating macrophages were sorted out by FACS and assessed for p38 phosphorylation as in **f**. See Supplementary Fig. 5F for full unedited blots. **h** *Left panel*: Hematoxylin/eosin staining of the gastrocnemius of mice of both genotypes injected with CTX along with either PBS or 15 μg/g of anisomycin during 60 h. Captures are representative of four mice per genotype and condition. Scale bar: 500 μm. *Right panel*: Quantification of all mice as in the left panel. **i** *Left panel:* As in **h**, but muscle damage was assessed by EBD staining injected 9–12 h before euthanasia. Captures are representative of four mice per genotype and condition. Scale bar: 500 μm. *Right panel:* Quantification of EBD^+^ areas for all mice in the left panel. **j** *Left panel*: As in **i**, but muscles were examined for CD206 expression. Captures are representative of four mice per genotype and condition. Scale bar: 50 μm. See Supplementary Fig. 5G for individual staining. *Right panel:* Quantification of CD206^+^ cells for all mice in the left panel. **k** Macrophages from both genotypes labeled with CFSE were injected into the gastrocnemius of 6-month-old mdx;*Zeb1* (+/+) mice. Nine hours before euthanasia mice were also injected with EBD to assess muscle damage. Muscles were harvested 2 days after macrophage transplant. *Left panel*: Representative merged pictures of at least five mice per genotype. See Supplementary Fig. 5I for individual staining captures. Scale bar: 50 μm. *Right panel:* Quantification of EBD^+^ areas associated to CSFE-labeled macrophages for all mice as in the left panel. **l** As in **k**, mdx muscles transplanted with macrophages of either genotype were analyzed for gene expression by qRT-PCR. Data are the mean of at least four mice per condition. **m** *Left panel*: Scheme of the experiment. *Right panel*: Macrophages isolated from wild-type and *Zeb1* (+/−) gastrocnemius injected with CTX and either PBS or anisomycin were assessed for in vitro cytotoxicity on C2C12 myotubes. Macrophages originated from at least three mice per genotype and condition

In line with Fig. 2a, e, dystrophic and acutely injured *Zeb1* (+/−) muscles contained a higher absolute number of infiltrating F4/80^+^ cells than wild-type muscles (Fig. 5a, b). FACS analysis of macrophage populations (CD11b^+^ F4/80^+^) isolated from the gastrocnemius of mice of both genotypes 2 days after CTX injection showed that the share of Ly6C^high^ pro-inflammatory macrophages was higher in *Zeb1* (+/−) muscles (Fig. 5c d and Supplementary Fig. 5a). In turn, the share of eosinophils [CD45b^+^ CD11b^+^ CD170^+^(SIGLECF^+^)]—which also participate in muscle injury and regeneration (e.g. ref. ^48^)—was similar in wild-type and *Zeb1* (+/−) injured muscles (Supplementary Fig. 5b, c). The upregulation of the anti-inflammatory macrophage marker CD206 (MRC1) was retarded in *Zeb1* (+/−) CTX-injured muscles (Fig. 5e and Supplementary Fig. 5d). Altogether these data indicate that ZEB1 not only inhibits muscle infiltration by pro-inflammatory macrophages upon injury but that ZEB1 is also required for their switch toward an anti-inflammatory phenotype.

The transition of pro-inflammatory macrophages toward an anti-inflammatory stage is regulated by the degree of activation of the p38-MAPK—that reaches its phosphorylation peak at day 2–3 post-injury—and its balance with DUSP1 (MKP-1), both a p38 target and its own inactivating phosphatase^17^. Forty eight hours after the CTX injection, and compared to wild-type counterparts, the phosphorylation of p38 in *Zeb1* (+/−) macrophages was greatly reduced (Fig. 5f, *left panel* and Supplementary Fig. 5e) and, accordingly, it was accompanied by lower levels of *Dusp1* (Fig. 5f, *right panel*).

We then investigated whether deficient p38 activation in *Zeb1* (+/−) macrophages accounts for the enhanced infiltration, delayed macrophage transition, and greater damage found in *Zeb1* (+/−) injured muscles. The gastrocnemius of wild-type and *Zeb1* (+/−) mice were injected with CTX along with anisomycin, a potent p38 activator^49^. At day 2.5 post-CTX injection, p38 is phosphorylated at maximum levels in wild-type macrophages and, consequently, anisomycin did not induce further activation. However, anisomycin efficiently phosphorylated p38 in *Zeb1* (+/−) macrophages to similar levels than that in wild-type macrophages (Fig. 5g and Supplementary Fig. 5f). Interestingly, anisomycin reverted the enhanced immune cell infiltration and tissue damage in *Zeb1* (+/−) muscles to similar levels than in wild-type ones (Fig. 5h, i). Likewise, anisomycin accelerated the transition of *Zeb1* (+/−) macrophages to an anti-inflammatory status (Fig. 5j and Supplementary Fig. 5g). Noteworthy, the failure of anisomycin to induce further p38 phosphorylation in wild-type macrophages at day 2.5 post-CTX injection correlated with its lack of effect on immune cell infiltration, tissue damage, or macrophage transition in wild-type muscles (Fig. 5g–j). Altogether, these results indicate that the deficient activation of p38-MAPK in *Zeb1* (+/−) muscles accounts for their greater immune cell infiltration and enhanced damage after injury.

Regulatory T (Treg) (CD4^+^ FOXP3^+^) cells accumulate in acutely injured muscles and limit macrophage response to IFN-γ^40,50–52^. Although the depletion of Treg cells in CTX-injured mice yields a phenotype that resembles that found in *Zeb1* (+/−) injured muscles^52^, we found no significant difference between the share of TReg cells in wild-type and *Zeb1* (+/−) injured muscles (Supplementary Fig. 5h).

### *Zeb1* (+/−) macrophages produce greater muscle damage than wild-type counterparts

The *Zeb1* (+/−) mouse model does not allow to examine the effect in muscle damage of downregulating *Zeb1* specifically in macrophages. Therefore, we tested whether isolated *Zeb1* (+/−) macrophages can cause greater damage than wild-type counterparts in a cell-autonomous manner. Wild-type and *Zeb1* (+/−) macrophages labeled with the 5(6)-Carboxyfluorecein diacetate N-succinimidyl ester (CFSE) fluorescent tracer were injected into the gastrocnemius of mdx;*Zeb1* (+/+) mice whose damage was then assessed by EBD uptake. Examination of CFSE^+^ macrophage infiltrated areas revealed that *Zeb1* (+/−) macrophages induced greater muscle damage than wild-type ones (Fig. 5k and Supplementary Fig. 5i). Muscles injected with *Zeb1* (+/−) macrophages expressed higher levels of pro-inflammatory markers than those that had received wild-type macrophages (Fig. 5l).

Additionally, *Zeb1* (+/−) macrophages displayed greater cytotoxic activity on myotubes than wild-type counterparts (Fig. 5m). This enhanced cytotoxicity of *Zeb1* (+/−) macrophages was reduced to similar levels than that of wild-type macrophages when CTX-injured muscles were simultaneously injected with anisomycin (Fig. 5m), supporting that the deficient phosphorylation of p38 in *Zeb1* (+/−) macrophages is responsible for the greater damage found in *Zeb1* (+/−) injured muscles.

It can be, therefore, concluded that *Zeb1* (+/−) macrophages are intrinsically capable—both in vitro and in vivo, and independently of the host background—of inducing greater tissue damage. These results also indicate that full levels of ZEB1 in the infiltrating macrophages, not only in myofibers, protect muscle from damage.

### ZEB1 is required for efficient muscle regeneration upon injury

**Fig. 6:**
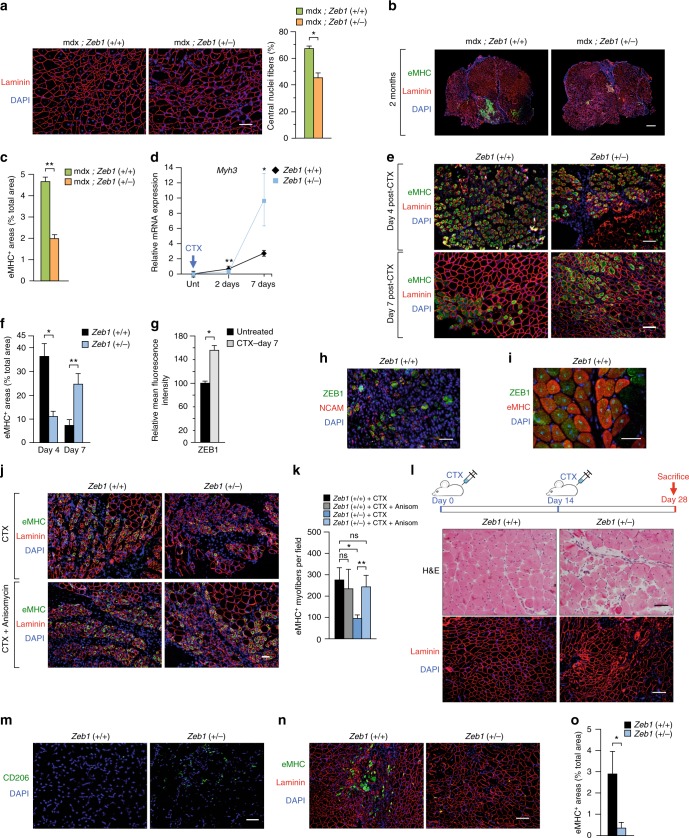
ZEB1 is required for efficient muscle regeneration upon injury. **a** *Left panel*: The gastrocnemius of 2-month-old mdx;*Zeb1* (+/+) and mdx;*Zeb1* (+/−) mice were stained for laminin (4H8-2) and DAPI. *Right panel*: Quantification of the percentage of centralized nuclei in the left panel for at least four mice per genotype. Scale bar: 100 μm. **b** As in **a**, but muscles were stained for eMHC (F1.652), laminin (4H8-2), and DAPI. Representative pictures from at least six mice per genotype. Scale bar: 500 μm. **c** Quantification of eMHC^+^ areas in **b**. Data are the mean of six mice per genotype. **d** *Myh3* mRNA levels were determined by qRT-PCR in the gastrocnemius of wild-type and *Zeb1* (+/−) mice either untreated or at day 2 and 7 after CTX injection. Data are the mean of at least three mice per genotype. **e** As in **d**, but wild-type and *Zeb1* (+/−) muscles were stained for eMHC (F1.652), laminin (4H8-2), and DAPI 4 or 7 days after CTX injection. Pictures are representative from at least four mice per genotype. Scale bar: 100 μm. **f** Quantification of eMHC^+^ areas in **e** from at least four mice per genotype. **g** The gastrocnemius of wild-type muscles either untreated or 7 days after CTX injection were assessed for ZEB1 (H-102) expression—quantified as mean fluorescence intensity (MFI)—in regenerating eMHC^+^ myofibers. Data are the mean of at least three mice. **h** Four days after CTX injection, wild-type gastrocnemius were stained for ZEB1 (H-102), NCAM (AF2408), and DAPI. Representative merged pictures from three mice. See Supplementary Fig. 6A for single staining. Scale bar: 50 μm. **i** As in **h**, but wild-type gastrocnemius were stained for ZEB1 (H-102), eMHC (F1.652), and DAPI 7 days after CTX injection. Representative merged pictures from three mice. See Supplementary Fig. 6B for single staining captures. Scale bar: 50 μm. **j** As in **e**, but wild-type and *Zeb1* (+/−) gastrocnemius were stained for eMHC 3 days after being injected with CTX and anisomycin. Representative merged pictures from four mice per genotype and condition. Scale bar: 50 μm. **k** Quantification of eMHC^+^ fibers per field. Five independent fields at x20 were assessed from of all mice in **j**. **l** The gastrocnemius of wild-type and *Zeb1* (+/−) mice were injected with two rounds of CTX and 14 days after they were stained for hematoxilin/eosin (H&E) (*upper panel*) or for laminin (4H8-2) and DAPI (*lower panel*). Representative captures from four mice per genotype. See Supplementary Fig. 6C for additional H&E captures. Scale bars for H&E and immunofluorescence pictures represent 50 and 100 μm, respectively. **m** As in **I**, but gastrocnemius were stained for CD206 (MR5D3) and DAPI.. Representative merged pictures from at least four mice per genotype. See Supplementary Fig. 6D for single staining. Scale bar: 50 μm. **n** Gastrocnemius muscles as in **I** were stained for eMHC, laminin, and DAPI as in **e**. Scale bar: 100 μm. **o** Quantification of eMHC^+^ areas out of the total tissue section area in **n**. Data are the mean from four mice per genotype

ZEB1 expression in the centralized nuclei of dystrophic muscles (Fig. 1b, d, e) prompted us to investigate a potential role of ZEB1 in muscle repair. The number of centralized nuclei in mdx;*Zeb1* (+/−) muscles was reduced by a third with regard to mdx;*Zeb1* (+/+) counterparts (Fig. 6a). The area stained for eMHC—a hallmark of regeneration in adult muscle^53^—in 2-month-old mdx;*Zeb1* (+/+) mice doubled that in mdx;*Zeb1* (+/−) muscles (Fig. 6b, c), suggesting that *Zeb1* downregulation resulted in poorer regeneration of dystrophic mdx muscles.

The role of ZEB1 in the regulation of eMHC during muscle regeneration was also examined in CTX-induced acute injury. The expression of *Myh3* (the gene encoding eMHC) mRNA and eMHC protein at days 2 and 4 after CTX injection, respectively, were lower in *Zeb1* (+/−) muscles than in wild-type muscles (Fig. 6d–f). Tissue damage in wild-type muscles has been repaired to a large extent by day 7 post-CTX; however, *Zeb1* (+/−) muscles still showed wide areas of infiltration and early regeneration (Fig. 2e). Accordingly, 7 days after CTX injection *Myh3* and eMHC were higher in *Zeb1* (+/−) muscles than in wild-type ones (Fig. 6d–f), indicating that muscle regeneration is retarded in *Zeb1* (+/−) mice.

ZEB1 increased in wild-type eMHC^+^ regenerating myofibers and was coexpressed with NCAM (CD56)—another marker of muscle regeneration^54,55^—and eMHC at days 4 and 7 post-CTX, respectively (Fig. 6g–i and Supplementary Fig. 6a, b).

The p38-dependent transition of macrophages toward an anti-inflammatory state modulates the timing of MuSC differentiation in injured muscles^1,3,7,13,17^. We found that anisomycin increased eMHC expression in *Zeb1* (+/−) muscles at day 3 post-CTX injection up to similar levels than in wild-type muscles (Fig. 6j, k) suggesting that deficient p38 phosphorylation in *Zeb1* (+/−) macrophages not only contributes to the enhanced immune infiltration and tissue damage in *Zeb1* (+/−) injured muscles (Fig. 5h–j) but, at least in part, also to their poorer regeneration.

The above results cannot exclude that the delayed and poorer muscle repair in *Zeb1* (+/−) muscles is also due to their intrinsically deficient MuSCs. To examine this, we challenged muscle regenerative capacity by administering two rounds of acute injury^56^ (Fig. 6l). Fourteen days after the second CTX injection, wild-type muscles still harbored myofibers with centralized nuclei but they had already recovered a relatively normal histological structure (Fig. 6l and Supplementary Fig. 6c). In contrast, *Zeb1* (+/−) muscles still displayed areas with abundant immune cell infiltration, including CD206^+^ anti-inflammatory macrophages that were not found in wild-type muscles (Fig. 6l, m and Supplementary Fig. 6C, D). *Zeb1* (+/−) muscles also exhibited impaired regeneration with myofibers of heterogenous size and aberrant shapes and lower expression of eMHC (Fig. 6l, n, o). In sum, while after a single injury insult *Zeb1* (+/−) muscles were able to fully regenerate albeit displaying a delayed repair (Fig. 2e), a second injury further compromised their regeneration, thus suggesting that *Zeb1* (+/−) MuSCs are functionally deficient.

### MuSCs require full levels of ZEB1 to maintain their quiescence

The activation and myogenic progression of MuSCs is determined by a well-defined gene signature^1–3,57^. Quiescent MuSCs express PAX7 but not MYOD1 (PAX7^+^MYOD1^–^) and do not incorporate BrdU. When MuSCs are activated by injury or stress, they uptake BrdU and gain MYOD1 expression (PAX7^+^MYOD1^+^), being then referred as adult myoblasts or muscle progenitor cells. Only after myoblasts have exited the cell cycle and differentiate do they lose PAX7 (PAX7^–^ MYOD1^+^). Lastly, they acquire MYOG, initially in coexpression with MYOD1^1^.

We first examined by immunofluorescence the expression of ZEB1 and/or PAX7 in wild-type muscles. In the absence of injury, most ZEB1^+^ nuclei were negative for PAX7, only 11.3% were also positive for PAX7 (Supplementary Fig. 7a). In turn, among PAX7^+^ nuclei, 78.1% were co-stained for ZEB1 for 21.9% that did not (Supplementary Fig. 7a). These data indicate that only a small fraction of ZEB1^+^ peripheral nuclei in Fig. 1b, c were MuSCs while most MuSCs expressed ZEB1.

**Fig. 7:**
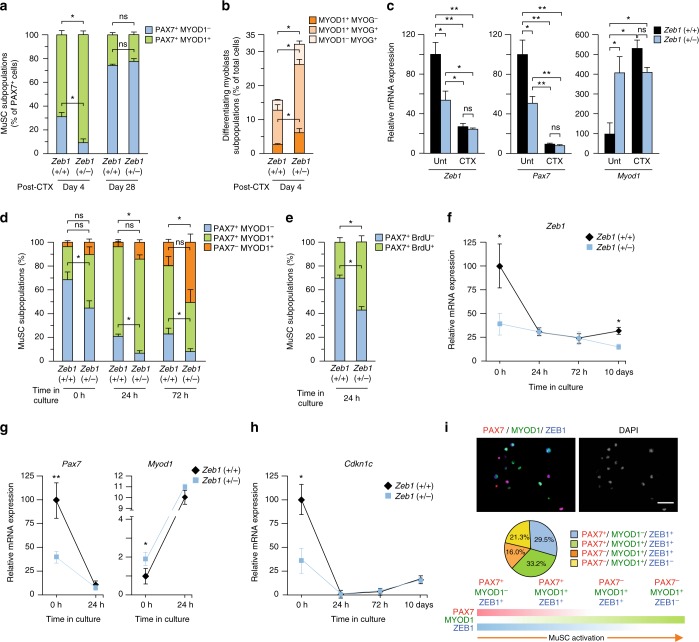
MuSCs require full levels of ZEB1 to maintain their quiescence. **a** The gastrocnemius muscles of wild-type and *Zeb1* (+/−) mice were injected with CTX and 4 or 28 days later, muscle sections were characterized for PAX7 (DSHB) and/or MYOD1 (C-20) expression. Data represent the percentage of each subpopulation out of total PAX7^+^ cells. Five independent fields at x40 from at least three mice per genotype were quantified. **b** Wild-type and *Zeb1* (+/−) injured gastrocnemius were assessed for MYOD1 (C-20) and MYOG (G-20) 4 days after CTX. Data represent the percentage of each subpopulation out of the total number of DAPI stained nuclei. Five independent fields at x40 from at least three mice per genotype were quantified. **c** MuSCs isolated by FACS from wild-type and *Zeb1* (+/−) mice either untreated or 48 h after CTX injection were examined for *Zeb1, Pax7*, and *Myod1* by qRT-PCR. Data are the mean of at least three mice per genotype. See Supplementary Fig. 7B–D for the sorting strategy. **d** MuSCs isolated by FACS from wild-type and *Zeb1* (+/−) mice were immunostained for PAX7 (DSHB) and MYOD1 (C-20) at the time of isolation and upon activation by ex vivo culture for 24 and 72 h. See Supplementary Fig. 7E for representative captures. Data are the mean of at least four mice per genotype. **e** As in **d**, but MuSCs were characterized for their PAX7 (DSHB) and/or BrdU uptake after 24 h of ex vivo culture. See Supplementary Fig. 7F for representative captures. **f**–**h** MuSCs isolated by FACS from wild-type and *Zeb1* (+/−) mice were analyzed for mRNA levels of the indicated genes by qRT-PCR at the time of isolation, as well as during their activation by ex vivo culture. Data are the average of least four mice per genotype. **i** *Upper panel:* Wild-type MuSCs isolated by FACS were cultured for 72 h and stained for ZEB1 (E-20), PAX7 (DSHB), and MYOD1 (C-20). See Supplementary Fig. 7J for individual staining and additional staining combinations. Scale bar: 50 μm. *Middle panel*: Distribution of the indicated wild-type MuSC subpopulations quantified from at least three mice as in the upper panel. *Lower panel:* PAX7, MYOD1, and ZEB1 expression during MuSC activation and myogenic conversion

Next, we explored whether ZEB1 expression modulates the in vivo distribution of PAX7^+^ subpopulations in response to muscle injury. At day 4 post-CTX injection, and compared to wild-type counterparts, the share of PAX7^+^ MYOD1^–^ cells was lower in *Zeb1* (+/−) muscles while that of PAX7^+^ MYOD1^+^ proliferating myoblasts was higher (Fig. 7a). However, 28 days after injury, the two PAX7^+^ fractions were similar in both genotypes (Fig. 7a). These results indicate that injury activated a larger share of MuSCs in *Zeb1* (+/−) muscles than in wild-type ones but the former were eventually able to recover their pool of quiescent MuSCs (PAX7^+^ MYOD1^–^) just as wild-type muscles did.

Muscles from both genotypes were also stained for MYOD1 and/or MYOG at day 4 post-CTX injection. Compared to wild-type counterparts, the share of differentiating myoblasts was higher in *Zeb1* (+/−) muscles (Fig. 7b), indicating that ZEB1 inhibits MuSC myogenic conversion while its downregulation drives MuSCs towards a differentiating stage.

Gene expression was also examined ex vivo in MuSCs isolated by FACS from wild-type and *Zeb1* (+/−) muscles either untreated or 2 days after CTX injection (Supplementary Fig. 7b–d). In untreated mice, *Zeb1* (+/−) MuSCs expressed lower levels of *Zeb1* and *Pax7* and higher of *Myod1* than wild-type MuSCs (Fig. 7c). As expected, CTX-induced injury activated MuSCs in both genotypes and caused the downregulation of *Pax7* and the upregulation of *Myod1* but, notably, injury brought the expression of all three genes in wild-type MuSCs to similar levels than in *Zeb1* (+/−) counterparts (Fig. 7c and see below).

Besides muscle injury, quiescent MuSCs can be also activated—and subsequently proliferate and differentiate—when cultured ex vivo^58,59^. Freshly isolated (0 h) wild-type MuSCs consist mainly (70–80%) of quiescent cells (PAX7^+^ MYOD1^–^), but they also contain a small subpopulation (around 15-25%) of PAX7^+^ MYOD1^+^ cells and <5% of PAX7^–^ MYOD^+^ cells (Fig. 7d and Supplementary Fig. 7e). In contrast, less than half of freshly isolated *Zeb1* (+/−) MuSCs were PAX7^+^ MYOD1^–^ while the majority exhibited an activated phenotype (PAX7^+^ MYOD1^+^) and the share of differentiating cells (PAX7^–^ MYOD^+^) was also larger than in wild-type counterparts (Fig. 7d and Supplementary Fig. 7e). As expected, ex vivo culture of wild-type MuSCs for up to 72 h reduced the share of PAX7^+^ MYOD1^–^ cells and increased that of PAX7^+^ MYOD1^+^ and PAX7^–^ MYOD^+^ cells. However, while differentiated myoblasts (PAX7^–^ MYOD1^+^) remained the smallest subpopulation in cultures of wild-type MuSCs after 72 h, this fraction represented the largest in *Zeb1* (+/−) MuSC cultures. Lastly, unike wild-type MuSCs, the majority of *Zeb1* (+/−) MuSCs after 24 h of ex vivo culture were PAX7^+^ BrdU^+^ (Fig. 7e and Supplementary Fig. 7f).

ZEB1 expression itself was also examined in MuSCs sorted by FACS. At the time of isolation, wild-type MuSCs expressed more than twice the levels of *Zeb1* than *Zeb1* (+/−) MuSCs (Fig. 7f). In line with in vivo MuSC activation by injury (Fig. 7c), activation of wild-type MuSCs by ex vivo culture downregulated *Zeb1* to similar levels than in freshly isolated *Zeb1* (+/−) MuSCs (Fig. 7f). When MuSCs from both genotypes were allowed to differentiate in vitro *Zeb1* mRNA levels remained relatively stable (Supplementary Fig. 7g) while *Myh4*—a marker of terminal muscle differentiation^53^—increased and was expressed at higher levels in *Zeb1* (+/−) MuSCs (Supplementary Fig. 7h). These data suggest that *Zeb1* downregulation primes MuSCs for both activation and myogenic conversion.

In line with Fig. 7c, at the time of their isolation by FACS *Zeb1* (+/−) MuSCs expressed lower levels of *Pax7* and higher of *Myod1* than wild-type counterparts (Fig. 7g). Ex vivo culture of wild-type MuSCs also downregulated *Pax7* to the same levels than those exhibited by *Zeb1* (+/−) MuSCs while *Myod1* increased in both genotypes (Fig. 7g), supporting our conclusion above that ZEB1 inhibits the premature activation of MuSCs. Freshly isolated wild-type MuSCs also expressed higher levels of the cyclin-dependent kinase inhibitor *Cdkn1c*/p57^KIP2^, which were downregulated upon ex vivo culture to similar levels than those in *Zeb1* (+/−) MuSCs (Fig. 7h). Given that M-cadherin (*Cdh15*) is expressed in both quiescent and activated MuSCs^55^, we also examined its expression in isolated wild-type and *Zeb1* (+/−) MuSCs along with that of E-cadherin (*Cdh1*) and N-cadherin (*Cdh2*). *Cdh2* and *Cdh15* were expressed at higher levels in *Zeb1* (+/−) MuSCs while *Cdh1* was undetectable in MuSCs from both genotypes (Supplementary Fig. 7i).

ZEB1 expression was also assessed in the different subpopulations of wild-type MuSC isolated by FACS. ZEB1 was found in both quiescent PAX7^+^ MYOD1^–^ MuSCs and activated PAX7^+^ MYOD1^+^ myoblasts, as well as in a fraction of differentiating PAX7^–^ MYOD1^+^ myoblasts (Fig. 7i and Supplementary Fig. 7j). This expression pattern suggests that ZEB1 is expressed in quiescent MuSCs but it is downregulated as MuSCs differentiate into myoblasts.

The conclusions from the above data are twofold. First, under basal conditions, wild-type MuSCs display a more quiescent signature than *Zeb1* (+/−) MuSCs that are already in a “primed” or “preactivated” stage. In other words, MuSCs require full levels of *Zeb1* to maintain their quiescence and a partial downregulation of *Zeb1* is sufficient to prompt their premature activation and myogenic progression in response to injury or following ex vivo culture. Second, activation of wild-type MuSCs by muscle injury or ex vivo culture brought their expression of *Pax7, Myod1, Cdkn1c*, and of *Zeb1* itself to similar levels than those in *Zeb1* (+/−) MuSCs.

### ZEB1 inhibits the positive feedback loop between MuSCs and macrophages

**Fig. 8:**
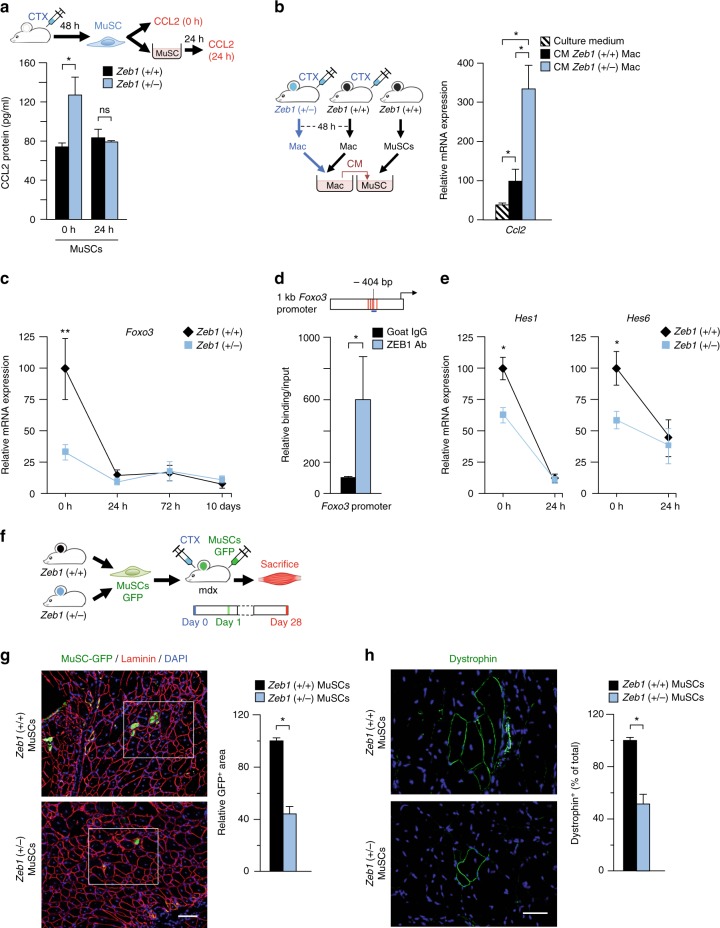
ZEB1 inhibits CCL2 and induces FOXO3 and HES1/HES6 in MuSCs and is required for MuSCs to drive muscle regeneration. **a** The gastrocnemius of wild-type and *Zeb1* (+/−) mice were injured with CTX and 48 h later their MuSCs were isolated by FACS and assessed for CCL2 at the time of isolation or from their conditioned medium (CM) after 24 h in culture. Data are the mean of at least three mice per genotype. **b** *Left panel*: Wild-type and *Zeb1* (+/−) gastrocnemius were injured with CTX and 48 h later their macrophages were isolated and cultured for 24 h. Wild-type MuSCs were then cultured for 24 h with either plain culture medium or with the CM collected from macrophages of either genotype. *Right panel*: *Ccl2* expression was assessed by qRT-PCR. Data are the mean of at least three mice per genotype and condition. **c** *Foxo3* mRNA levels were determined by qRT-PCR in wild-type and *Zeb1* (+/−) MuSCs at the time of isolation or after ex vivo culture for the indicated times. Data are the mean of least four mice per genotype. **d** *Upper panel*: Scheme of 1 kb of the mouse *Foxo3* promoter. Consensus binding sites for ZEB1 are marked as vertical red lines. The promoter region assessed by ChIP for a ZEB1 binding site at −404 bp (Supplementary Methods) is represented by a horizontal blue line. *Lower panel*: DNA from C2C12 myoblasts was immunoprecipitated with antibodies against ZEB1 (E-20) or control goat IgG and amplified by qRT-PCR for the indicated *Foxo3* promoter region. Data are the mean of four independent experiments. **e** Wild-type and *Zeb1* (+/−) MuSCs were assessed for *Hes1* and *Hes6* mRNA levels by qRT-PCR either at the time of isolation or after 24 h in culture. Data are the mean of at least four mice per genotype. **f** GFP-labeled wild-type and *Zeb1* (+/−) MuSCs (see Supplementary Fig. 8A) were transplanted into the gastrocnemius of mdx;*Zeb1* (+/+) mice that had been injected 24 h earlier with CTX. Four weeks later mice were euthanized and their gastrocnemius assessed for muscle regeneration. **g** *Left panel*: As in **f**, muscle regeneration was evaluated by the presence of GFP^+^ myofibers assessed by immunostaining for GFP (GFP-1020), laminin (4H8-2), and DAPI. See Supplementary Fig. 8B for higher magnification captures and individual staining of the inset area. Representative captures of at least four mice per genotype. Scale bar: 100 μm. *Right panel*: Quantification of areas stained for GFP as in the left panel. **h** As in **g**, but regeneration was assessed by immunostaining for dystrophin (MANDRA-1). *Left panel*: representative captures from at least four mice per genotype. Scale bar: 100 μm. *Right panel*: Quantification of areas stained for dystrophin as in the left panel

Activated MuSCs secrete CCL2 to promote monocyte chemotaxis into injured muscles^6^. Figure 4c, e showed that CTX-injured *Zeb1* (+/−) total muscles and isolated myofibers produced more CCL2 than wild-type counterparts. MuSCs isolated from *Zeb1* (+/−) CTX-injured muscles also produced more CCL2 than MuSCs isolated from wild-type peers (Fig. 8a), supporting the enhanced macrophage infiltration in *Zeb1* (+/−) muscles upon injury. As for other genes (Fig. 7g, h), CCL2 secretion by MuSCs became similar in both genotypes upon ex vivo culture (Fig. 8a).

We next explored the other side of the macrophage-MuSC crosstalk and investigated whether wild-type and *Zeb1* (+/−) macrophages have a differential effect on CCL2 production by MuSCs. Wild-type MuSCs were incubated with plain culture medium or with the conditioned medium (CM) produced by macrophages isolated from wild-type and *Zeb1* (+/−) CTX-injured muscles. Compared to plain medium, the CM collected from wild-type and *Zeb1* (+/−) macrophages increased *Ccl2* in wild-type MuSCs but this upregulation was much larger with the CM from *Zeb1* (+/−) macrophages (Fig. 8b).

Collectively, data in Fig. 8a, b indicate that ZEB1 expression in MuSCs and macrophages has an inhibitory effect on both sides of the positive feedback loop existing between these cell types and that promotes macrophage migration upon injury.

### MuSCs depend on ZEB1 to induce FOXO3 and NOTCH targets, and to drive muscle regeneration

FOXO3 is required for MuSCs self-renewal during muscle regeneration^56^. *Foxo3* (−/−) muscles exhibit retarded regeneration in response to injury and their MuSCs are unable to maintain quiescence^56,60^. As for *Pax7* (Fig. 7g), *Foxo3* mRNA levels in freshly isolated wild-type MuSCs were about twice of those in *Zeb1* (+/−) counterparts but ex vivo culture reduced them to those in *Zeb1* (+/−) MuSCs (Fig. 8c). Examination of the mouse *Foxo3* promoter revealed the existence of several high-affinity consensus binding sites for ZEB1 and ChIP assays confirmed ZEB1 binding to the *Foxo3* promoter (Fig. 8d).

Notch signaling maintains MuSC quiescence by repression of *Myod1* either directly through Notch target genes of the HES and HEY families or indirectly via *Pax7* activation^57,61–63^. In line with their premature activation, *Zeb1* (+/−) MuSCs exhibited lower expression of *Hes1* and *Hes6* than wild-type counterparts (Fig. 8e).

Results in Fig. 6l suggested that poorer regeneration of *Zeb1* (+/−) injured muscles is related, at least in part, to their intrinsically deficient MuSCs. As the *Zeb1* (+/−) mouse model does not allow examining the effect in muscle regeneration of downregulating *Zeb1* specifically in MuSCs, isolated wild-type and *Zeb1* (+/−) MuSCs were transplanted into the same recipient background—the mdx;*Zeb1* (+/+) mouse—and compared in their regenerative capacity. Exogenous injury of mdx dystrophic muscles prior to MuSC transplant enhances engraftment and muscle regeneration^64^. Thus, GFP-labeled MuSCs isolated from both genotypes were injected into the gastrocnemius of CTX-injured mdx;*Zeb1* (+/+) mice (Fig. 8f and Supplementary Fig. 8a). Four weeks later, mice were euthanized and the presence of myofibers positive for GFP or dystrophin—for which mdx muscles are deficient—were assessed by immunofluorescence as proxies of newly formed myofibers derived from the transplanted MuSCs. Through both approaches, it was found that wild-type MuSCs generated about twice as many more new myofibers than *Zeb1* (+/−) MuSCs (Fig. 8g, h and Supplementary Fig. 8b). From these data, it can be concluded that MuSCs require full levels of ZEB1 to drive efficient muscle regeneration.

## Discussion

**Fig. 9:**
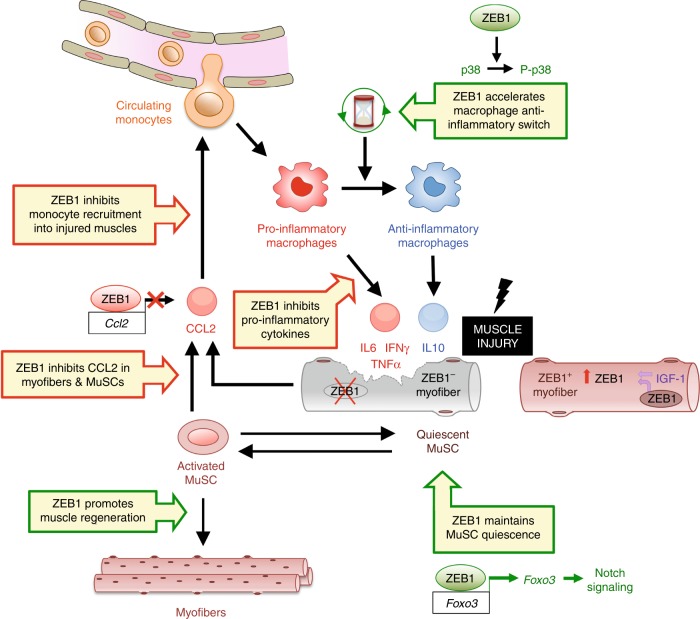
ZEB1 protects skeletal muscle from damage and is required for its regeneration. See main text for details

Muscle injury and regeneration are closely linked processes as the latter depends on the precise and timely sequencing of the pro-inflammatory and anti-inflammatory signals that occur in the context of muscle damage^11,17^. The molecular mechanisms that coordinate muscle injury and regeneration are only partially understood. Here, we show that ZEB1 protects muscle from damage and is required for its regeneration (Fig. 9).

The role of ZEB1 in muscle injury and regeneration uncovered here occurs at multiple levels and in different cell types. Monocytes/macrophages, the predominant immune cell that infiltrates damaged muscles, are recruited from pro-inflammatory circulating monocytes by CCL2 and other chemokines produced by damaged myofibers, activated MuSCs, and infiltrated macrophages themselves^6,7,9,10,13^. We found that ZEB1 has an inhibitory effect on both sides of the positive feedback loop between MuSCs and macrophages that promotes macrophage infiltration upon injury, loop that is amplified in *Zeb1* (+/−) muscles. Thus, on the one hand, ZEB1 directly repressed the *Ccl2* promoter and, accordingly, *Zeb1* (+/−) myofibers and MuSCs produced higher levels of CCL2 than wild-type counterparts. On the other hand, soluble factors secreted by macrophages of both genotypes, but at higher levels by *Zeb1* (+/−) ones, stimulated CCL2 production by MuSCs.

ZEB1 not only inhibited CCL2-mediated macrophage infiltration of injured muscles but its expression in macrophages suppressed their cytotoxic activity on muscle cells and accelerated their transition toward an anti-inflammatory status. Thus, *Zeb1* (+/−) macrophages were intrinsically capable of producing greater damage than wild-type ones and produced higher levels of pro-inflammatory cytokines. The switch of pro-inflammatory macrophages first toward an anti-inflammatory phenotype and later to a cytokine exhaustion stage is driven by the level of phosphorylation of p38-MAPK and its balance with DUSP1^17^. In response to injury, *Zeb1* (+/−) macrophages displayed deficient p38 activation and, at least for the timeframe examined, lower DUSP1 levels. Anisomycin efficiently phosphorylate p38 in *Zeb1* (+/−) macrophages and reverted the enhanced infiltration and damage in *Zeb1* (+/−) injured muscles to the same levels than in wild-type counterparts. Reduced *Dusp1* expression in *Zeb1* (+/−) muscles suggests that their deficient p38 activation following injury is not due to its de-phosphorylation by DUSP1. Interestingly, p38 signaling is required for TGFβ-induced EMT^65^ and we are currently investigating the precise mechanism by which ZEB1 stimulates p38 phosphorylation.

ZEB1 also promoted muscle repair through, at least, two independent mechanisms. First, the delayed and poorer regeneration of *Zeb1* (+/−) injured muscles was related to the retarded transition of their macrophages towards an anti-inflammatory stage. Accordingly, forced p38 phosphorylation in *Zeb1* (+/−) muscles by anisomycin increased the number of eMHC^+^ regenerating myofibers to similar levels than in wild-type muscles. It is possible that the improved muscle regeneration induced by anisomycin results from a direct effect in MuSCs. However, p38 signaling inhibits MuSC expansion in mdx mice while its genetic ablation or pharmacological blockade expands MuSCs and increases the number of activated MuSCs^14,66,67^. Additionally, the defective muscle regeneration in *Dusp1* (−/−) mice depends on the alteration of the p38/DUSP1 balance in macrophages, not in MuSCs, as it is rescued by bone marrow transplantation^17^.

Second, poorer regeneration in *Zeb1* (+/−) muscles was also related to their intrinsically defective MuSCs. MuSCs required full homeostatic levels of ZEB1 to mount an efficient engraftment upon transplantation in dystrophic muscles. Data indicated that *Zeb1* (+/−) MuSCs are already “primed” towards an activated/differentiated stage. Early during activation, *Zeb1* (+/−) MuSCs contained a smaller share of quiescent cells and a higher fraction of proliferating and differentiating myoblasts than the activation of wild-type MuSCs. Expression of *Zeb1* and of quiescence-associated genes (*Pax7, Foxo3, Hes1, Hes6)* in freshly isolated *Zeb1* (+/−) MuSCs was between a third and a half of those found in wild-type MuSCs. Interestingly, activation of wild-type MuSCs downregulated the expression of all these genes to the same levels than those found in *Zeb1* (+/−) MuSCs at the time of isolation. ZEB1’s role maintaining MuSC quiescence seems to depend on a gene expression threshold of these quiescence-associated genes and of ZEB1 itself below which MuSCs become primed toward an accelerated activation and differentiation. Of note, ZEB1 is upregulated in cancer and tumor microenvironment cells but its downregulation to around half of the original upregulated levels is sufficient to block tumor progression^28–30^.

MuSCs depend on ZEB1 for their expression of several quiescence-associated genes suggesting that ZEB1 acts through a common upstream regulator. Notch signaling maintains MuSC quiescence by both direct and indirect inhibition of MYOD1^61–63^. We showed here that ZEB1 inhibits *Myod1* expression and it has been reported that ZEB1 represses MYOD1 transcriptional activity, and displace MYOD1 from its DNA binding sites on target genes^19,21,68^. FOXO3 supports MuSC quiescence through Notch signaling^56^ and, alternatively, ZEB1 may induce a quiescence-associated signature in MuSCs via its direct activation of the *Foxo3* promoter found here. Like in *Zeb1* (+/−) mice, *Foxo3* (−/−) muscles exhibit delayed regeneration that is severely impaired after two rounds of injury but not after a single challenge^56,60^.

Current therapies in muscular dystrophies aim at modulating the inflammatory response and improving the regenerative capacity of MuSCs. Our results here established ZEB1 as an important factor in the regulation of both processes, thus potentially opening new avenues in the treatment of muscular dystrophies.

## Methods

### Mouse and human samples

The source of mouse and human samples is detailed in Supplementary Methods. The use of animals in this study followed the guidelines of the Animal Experimentation Ethics Committee at the University of Barcelona (Barcelona, Spain) and was approved under reference UB/385/17. All human samples were obtained with the informed consent of patients, conformed to the principles of the Helsinki Declaration, and their use was approved by the Clinical Ethics Research Committee at Hospital Clinic of Barcelona (Barcelona, Spain) under reference HCB/17/0815.

### Antibodies, and DNA and RNA oligonucleotides

The antibodies used in the immunostaining, and in the isolation and characterization of immune cells and MuSCs are detailed in Supplementary Methods. DNA oligonucleotides used as primers in quantitative real time PCR (qRT-PCR) and RNA oligonucleotides used in RNA interference are described in Supplementary Methods.

### Immunostaining

The immunohistochemistry and immunofluorescence staining of mouse and human tissue samples, as well as cross-section area (CSA) analysis of muscle sections are described in Supplementary Methods.

### Cell surface protein expression and cell sorting by FACS

Characterization of infiltrating immune cells in injured muscles and isolation of macrophages and MuSCs from injured and/or non-injured muscles were performed by FACS as described in Supplementary Methods.

### Gene and protein expression

RNA extraction and subsequent analysis of gene expression by qRT-PCR, transcriptional analyses of promoter activity with luciferase reporters, chromatin immunoprecipitation (ChIP) assays, and assessment of protein expression by western blot are described in Supplementary Methods.

### Characterization and transplant of macrophages and MuSCs

The phenotypic and functional characterization of macrophages and MuSCs, as well as their adoptive transfer into mdx mice are described in Supplementary Methods.

### Statistical analysis

Statistical analysis of data shown was performed using Prism for Mac version 5.0a (GraphPad Software Inc., La Jolla, CA, USA). Statistical significance was assessed with a two-sided non-parametric Mann–Whitney *U*-test. Correlations were assessed by the Spearman correlation test. Error bars in histograms represent standard errors of means. Relevant comparisons were labeled as either significant at *p* ≤ 0.001 (***), *p* ≤ 0.01 (**) or *p* ≤ 0.05 (*) levels, or non-significant (ns) for values of *p* > 0.05.

## Supplementary information

Supplementary Information

## Data Availability

All relevant data are available from the authors.
