# Supplementary material for: ZEB1 protects skeletal muscle from damage and is required for its regeneration
Source: Nat Commun. 2019 Mar 25;10:1364. doi: 10.1038/s41467-019-08983-8 (PMC6434033; doi:10.1038/s41467-019-08983-8)
Supplement: Supplementary file 1 — Supplementary Information [file 41467_2019_8983_MOESM1_ESM.pdf]

SUPPLEMENTARY DATA

ZEB1 protects skeletal muscle from damage and is required for its regeneration

Laura Siles [1], Chiara Ninfali [1*], Marlies Cortes [1*], Douglas S. Darling [2], Antonio Postigo [1,3,4 #]

[1] Group of Transcriptional Regulation of Gene Expression, Dept. of Oncology and Hematology. IDIBAPS, Barcelona 08036, Spain
[2] Center for Genetics and Molecular Medicine and Dept. of Immunology and Infectious Diseases, University of Louisville, KY 40202, USA
[3] Molecular Targets Program, James G. Brown Cancer Center, Louisville, KY 40202, USA
[4] ICREA, Barcelona 08010, Spain

* These authors contributed equally to this work

# Address correspondence to: A Postigo, Group of Transcriptional Regulation of Gene Expression. IDIBAPS. Casanova 143. 08036 Barcelona, Spain. Email: idib412@clinic.cat

<u>**SUPPLEMENTARY METHODS**</u>

Mouse models of muscle damage

The following mouse models were used: CL57BL/6 [referred throughout the article as either wild-type or *Zeb1* (+/+)] (Jackson Laboratories, Bar Harbor, ME), *Zeb1* (+/-) (25) and mdx [also referred throughout as mdx;*Zeb1* (+/+)] (Jackson Laboratories) (5). mdx and *Zeb1* (+/-) mice were crossed to generate *mdx;Zeb1* (+/-) mice. The mdx mouse was used as a model of muscular dystrophy and chronic muscle injury and specimens were male or homozygous females with the age indicated in each experiment. As a model of acute muscle injury the gastrocnemius muscle was injected with 50 µl of 10 µM cardiotoxin (CTX) from *Naja mossambica mossambica* (Sigma-Aldrich, St. Louis, MO). In experiments where mice were subjected to two rounds of acute injury, a second injection of CTX was administered 14 days after the first and mice were sacrificed 14 days later (see scheme in Fig. 6L). In selected experiments, damaged fibers were labeled *in vivo* with Evans Blue Dye (EBD) (Sigma-Aldrich, St. Louis, MO) as described elsewhere (15). Nine to twelve h before euthanasia, mice were injected intraperitoneally (i.p.) with 0.1 ml of a 10 mg/ml solution (in PBS) of EBD per 10 gr of total body weight. In experiments where anisomycin was used, gastrocnemius muscles were injected with 10 µM of CTX along with either PBS (left gastrocnemius) or with 15 µg/g of anisomycin (Sigma-Aldrich) (right gastrocnemius) maintaining the same volume and concentration of CTX for both gastrocnemius. Wherever indicated myofibers were isolated and maintained in culture as described elsewhere (21).

Human samples

A series of nine human muscle dystrophies (five Duchenne, four Becker) were obtained as slides of frozen tissue from University Hospital Complex of Vigo (Vigo, Spain) through the Spanish National Biobank Network. Clinical information associated to the cases included creatine kinase (CK) levels in serum.

C2C12 cell culture and differentiation

C2C12 cells were obtained from the American Type Culture Collection (ATCC-LGC Standards, Middlesex, UK) and were tested regularly to be negative for mycoplasma with e-Myco™ plus Mycoplasma PCR Detection kit (iNtRON Bio, Seongnam-si, South Korea). C2C12 cells were plated on 24-well plates and cultured in growth medium, namely, Dulbecco's modified Eagle Medium (DMEM) (Lonza, Basel, Switzerland) supplemented with 10% FBS (Sigma-Aldrich. St. Louis, MO), and 1% penicillin-streptomycin (Pen/Strep) (Lonza). When cells reached confluence, growth medium was replaced by differentiation medium, namely, DMEM supplemented with 2% of Horse serum (Sigma-Aldrich) and 1% Pen/Strep for an additional 24 hours. Cells were then incubated during 1 h in the presence or absence of 100 ng/ml of recombinant IGF-1 (PeproTech, reference 100-11, London, UK) and/or 20 µM of LY294002 (Sigma-Aldrich), and/or 40 µM of PD98059 (Sigma-Aldrich).

Determination of CCL2 protein levels

CCL2 protein production by total muscle or selected cells within—namely, myofibers, macrophages, and MuSCs—was quantified using a mouse CCL2 ELISA kit (Thermo Fisher Scientific, Waltham, MA) as per manufacturers' instructions. CCL2 was determined from cell lysates at the time of isolation (referred in the main text as 0 h) or from the supernatant of these cells upon culture in the specific respective media during 24 h.

Immunohistochemistry

Immunohistochemistry staining of muscles was performed as described elsewhere (24). Briefly, gastrocnemius muscles were dissected from mice, mounted on corks, embedded in Tissue-Tek® O.C.T. Compound (Electron Microscopy Sciences, Hatfield, PA), frozen in liquid

nitrogen-cooled isopentane, and stored at -80ºC. Next, 7 µm cryosections were prepared (Leica Cryostat CM 1950, Leica Biosystems, Wetzlar, Germany), fixed for 20 min in ice-cooled acetone and permeabilized in 0.25% Triton X-100 in PBS for 30 min. For each sample, at least one slide was routinely stained with hematoxylin and eosin. To block endogenous peroxidase, muscle samples slides were first incubated with 0.3% hydrogen peroxide in PBS and non-specific binding blocking solution (NSBBS) [5% donkey normal serum (Jackson Immuno-Research Europe Ltd., Cambridge- shire, England, UK), 4% BSA in PBS, 0.5% Tween 20]. Slides were then incubated overnight at 4°C with a ZEB1 antibody (H-102, 1/100 dilution) followed by an anti-rabbit HRP-conjugated secondary antibody during 1 h at room temperature. The immunohistochemistry reaction was developed with a DAB substrate kit (Vector Labs, Burlingame, CA) before slides were counterstained with hematoxylin and mounted in Di-N-butylPhthalate in Xylene solution (DPX, Sigma-Aldrich). Staining was evaluated in an Olympus BX41 microscope (Olympus, Tokyo, Japan) and images processed with ImageJ software (NIH, Bethesda, MD).

Immunofluorescence

For immunofluorescence staining of mouse muscles, gastrocnemius were embedded, frozen, cryo-sectioned and fixed as described above for immunohistochemistry. For immunofluorescence staining of C2C12 cells were fixed with 4% paraformaldehyde (Electron Microscopy Sciences, Hatfield, PA) for 20 min, and permeabilized with 0,25% Triton X-100 for 30 min. For the immunofluorescence staining of isolated muscle satellite cells (MuSCs) (see below), cells were pelleted, fixed in 4% paraformaldehyde and resuspended in gelatin. The resulting gelatin block of MuSCs was embedded in paraffin and sections were subjected to antigen retrieval for permeabilization. Fixed samples of mouse and human muscles, C2C12 cells, and MuSCs were then incubated with 0.1% $NaBH_4$ to block non-specific autofluorescence and with NSBBS to reduce non-specific antibody signal. Slides were then incubated with the corresponding primary (overnight at 4°C) and fluorochrome-conjugated secondary (1 h at room temperature) antibodies. In the immunofluorescence staining of mouse muscle sections with mouse primary antibodies MOM™ blocking reagent (Vector Laboratories) was added to the blocking buffer (1:40 dilution). The primary antibodies used in immunofluorescence (see Supplementary Table 1 and figure legends) were used at the following dilutions or concentrations from the commercially available product: CD4 (1/50), CD206 (1/20), dystrophin (1/80), F4-80 (10 µg/ml), FOXP3 (1/50), GFP (1/500), laminin (1/80), embryonic MHC (1/10), MYOD1 (1/50), MYOG (1/80), NCAM (10 mg/ml), PAX7 (undiluted hybridoma), and ZEB1 (1/100). Slides were then mounted with Prolong Gold® Antifade Reagent with DAPI (Thermo Fisher, Carlsbad, CA). Staining was evaluated in a Nikon Eclipse E600 microscope (Nikon, Tokyo, Japan) and images processed with ImageJ software. For the *in vivo* assessment of MuSC gene expression, five fields were counted for each condition and genotype. In selected experiments, the mean fluorescence intensity was assessed using ImageJ software in at least 50 myofibers from three mice.

Cross-sectional area analysis

Cross-sectional area (CSA) measurement of myofibers was performed on gastrocnemius samples stained for laminin by immunofluorescence using ImageJ software.

Flow cytometry analysis (FACS) and sorting of immune infiltrate

Gastrocnemius muscles were minced and digested in 0.05% pronase (Millipore, Billerica, Massachusetts), 2 U/ml collagenase Type I (Sigma-Aldrich) in DMEM (Lonza) at 37ºC during 1.5 h. Cell suspensions were then filtered through a 70 µm cell strainer (Thermo Fisher), centrifuged and erythrocytes were removed using Red Blood Lysis Buffer © (Sigma-Aldrich). Cells were then blocked for Fc receptors with mouse gamma globulin (Jackson ImmunoResearch Europe Ltd) and incubated in PBS with 2% FBS at 4ºC for 45 min with the corresponding fluorochrome-conjugated antibodies, namely: F4/80-APC (1/200), CD11b-PE (1/50), and Ly-6C-PerCP-Cy5.5 (1/150), CD45-PerCP Cy5.5 (1/200), CD3-APC (1/200), NK1.1-FITC (1/200), Ly6G-FITC (1/200), and CD170 (SIGLECF)-PerCP eFluor 710 (1/200). In

the characterization of eosinophils by SIGLECF out of the CD45+ CD11b+ population, cells from disaggregated muscles were first incubated with unconjugated CD45 (1/100) following by Alexa Fluor 647 goat anti-mouse (1/100), and CD11b-PE (1/50). See Supplementary Table 1 for the identity and source of these antibodies. Expression of cell surface markers was assessed in a BD FACSCanto™ II analyzer (BD Biosciences, San Jose, CA). The acquired data were then analyzed using FlowJo for Windows, version 7.6.1 (FlowJow, Ashland, OR). Macrophages were sorted out in a FACS Aria^{TM} II cell sorter (BD Biosciences) for subsequent analysis.

RNA extraction and qRT-PCR analysis

Total RNA from gastrocnemius muscle and isolated macrophages was extracted using TRIzol (Life Technologies, Thermo Fisher) while total RNA from freshly isolated and cultured MuSCs or macrophages was extracted with Arcturus® PicoPure® RNA Isolation Kit (Life Technologies, Thermo Fisher). RNA was retrotranscribed with random hexamers using High-Capacity cDNA Reverse Transcription kit (Life Technologies, Thermo Fisher). mRNA levels were determined by qRT-PCR using SYBRGreen GoTaq® qPCR Master Mix (Promega Corp, Madison, WI) in a Chromo4 real time PCR apparatus (Bio-Rad, Hercules, CA). Results were analyzed using Opticon Monitor 3.1.32 software (Bio-Rad) by the ΔΔCt method using *Gapdh* as reference gene. Primers used to amplify the different genes examined in the study are detailed in Supplementary Table 2.

Western blots

C2C12 cells, total muscles or isolated macrophages were resuspended in RIPA lysis buffer (150 mM NaCl, 50 mM Tris pH 8.0, 0.1% NP40, 0.5 % SDS, 2 mM EDTA) containing protease inhibitors (10 µg/ml aprotinin, leupeptin, pepstatin A, and PMSF) as previously described (24). Lysates were sonicated in a Sonics VibraCell CV188 instrument (Misonix Inc., Farmingdale, NY), clarified by centrifugation and quantified by Bradford assay. Lysates were then boiled and loaded onto 10 % polyacrylamide gels and transferred to a PVDF membrane (Immobilon-P, Millipore, Bedford, MA, USA). Membranes were blocked with 5 % non-fat milk and blotted with the corresponding primary antibodies overnight at 4ºC: α-tubulin (1:10,000), GAPDH (1:6,000), p38 (1/5,000), phosphorylated p38 (1/1,000), and ZEB1 (HPA027524) (1:500). Blots were then incubated with HRP-conjugated secondary antibodies and the luminescence reaction was developed with Clarity™ Western ECL (Bio-Rad). Images shown in the article are representative of at least three independent experiments. Full unedited blot images are included as Supplementary Figures with the area displayed in the main Figures boxed.

Chromatin Immunoprecipitation Assays

Chromatin immunoprecipitation (ChIP) assays were performed using EpiQuick ChIP kit (EpiGentek, Farmingdale, NY) as per manufacturer's instructions. Briefly, C2C12 cells were incubated for 30 min with 1% paraformaldehyde solution (Electron Microscopy) at room temperature followed by incubation with 1.25 mM glycine. Lysates were sonicated as described elsewhere (23). Goat anti-mouse/human ZEB1 (E-20) and its corresponding normal goat IgG (Jackson ImmunoResearch Europe Ltd.) were used. Identification of DNA binding sequences for ZEB1 in the *Ccl2* and *Foxo3* promoters and design of primers for qRT-PCR was conducted using MacVector software (MacVector Inc, Apex, NC). DNA fragments were quantified by qRT-PCR as detailed above using the primers detailed in Supplementary Table 3. In all qRT-PCRs, values shown represent relative binding in relation to input and are the average of at least four independent ChIP experiments, each performed in duplicate.

Transcriptional assays

Luciferase assays were performed using standard procedures. Briefly, C2C12 cells in growth medium (see above) and seeded in 12-well plates were transiently transfected with a firefly luciferase reporter for a 2 kb fragment of the *Ccl2* promoter, cDNA expression vectors and/or siRNAs (see Supplementary Table 4) with 2 µl of Lipofectamine 3000 (Thermo-Fisher) or

RNAiMAX (Thermo-Fisher), respectively, per well. As control, the corresponding expression empty vectors and/or control siRNA (siCtl) were also transfected. The total amount of transfected DNA was constant adjusting with pBluescript SK (Stratagene, Agilent Technologies, La Jolla, CA). One µg of a SV40-β-galactosidase vector (Promega Corp.) were used for normalization of transfection efficiency. Levels of luciferase and β-galactosidase activity were assayed 48 hours later with Luciferase Assay System kit (Promega Corp.) and Luminiscent β-galactosidase Detection kit II (Clontech, Takara Bio Inc., Kutasu, Siga-ken, Japan), respectively. Relative luciferase activity (RLU) was determined using Modulus II Glomax microplate reader (Promega Corp.). Data shown correspond to a representative experiment from at least four independent ones with each transfection conducted as duplicates. When RLU values are represented in a histogram, the control condition is set to a RLU value of 100.

Transplant of macrophages

Transplant of macrophages was conducted as described elsewhere (18). Wild-type and *Zeb1* (+/-) macrophages were generated from bone marrow total cells as described (29). Briefly, femur and tibia bone marrows from 6-8-weeks-old mice of both genotypes were flushed with PBS and collected cells were centrifuged and resuspended in DMEM supplemented with 10% FBS, and 1% penicillin-streptomycin and cultivated during 6 days with 20 ng/ml of recombinant CSF2 (Immuno Tools GmbH, Friesoythe, Germany). Every 2 days, half of the medium was replaced with fresh medium supplemented with CSF2. Macrophages were then incubated for 24 h with the tracer 5(6)-Carboxyfluorescein diacetate N-succinimidyl ester (CFSE) (Sigma-Aldrich). One million CFSE-labeled macrophages were centrifuged, resuspended in 50 ml of PBS and injected into the right hindlimb gastrocnemius of mdx;*Zeb1* (+/+) mice. Nine to 12 h before sacrifice mice were injected with EBD as described above. Two days later mice were euthanized and their gastrocnemius muscles were processed for immunofluorescence analysis.

Cytotoxicity assays

Macrophage-mediated cytotoxicity on C2C12 myotubes was determined using a protocol modified from the one described in (27). Briefly, C2C12 myoblasts were plated in 96-well plates and cultured in growth medium as described above. When cells reached confluence, the growth medium was replaced by differentiation medium for an additional 24 h to allow the formation of a confluent monolayer of C2C12 myotubes. Myotubes were then returned to complete medium for 48 h before being co-cultured with macrophages for cytotoxicity assays. The gastrocnemius of one hindlimb in wild-type and *Zeb1* (+/-) mice was injected with CTX while the contralateral gastrocnemius was injected with both CTX and anisomycin. After 48 h, mice were euthanized and the infiltrating macrophages from each gastrocnemius were isolated by sorting. A total of 1 x 10^5 macrophages of each genotype and condition were then plated on top of the monolayer of C2C12 myotubes and co-cultured in growth medium for 24 h. Macrophage cytotoxicity on myotubes was assessed by the release of lactose dehydrogenase (LDH) into the media from damaged myotubes using the Pierce™ LDH Cytotoxicity Assay Kit (Thermo Fisher) as per manufacturer's instructions. Cytotoxicity is expressed as a percentage of total myotube lysis, according to Equation 1:

Equation 1: $$\frac{\text{Abs M}\phi\ \text{MT} - \text{Abs M}\phi\text{ctrl} - \text{Abs MTctrl}}{\text{Abs MTlysis} - \text{Abs MTctrl}} \times 100$$

where Abs Mϕ MT is the experimental absorbance value for LDH release in the co-culture of macrophages and myotubes, Abs $M\phi_{ctrl}$ is the absorbance representing the spontaneous release of LDH by macrophages alone, Abs MT_{ctrl} is the absorbance corresponding to the spontaneous release of LDH by myotubes alone, and Abs MT_{lysis} represents the absorbance corresponding to the maximum LDH release by myotubes (100% lysis) determined by incubating myotubes with the kit's lysis buffer in growth medium for 1 h. Absorbance was

measured at 450 nm and 600 nm in a Modulus II Glomax microplate reader (Promega Corp.) Macrophages were isolated from at least three mice for each genotype and condition.

Phenotypic and functional characterization of MuSCs

MuSCs were isolated as previously described (21). Briefly, bilateral hindlimb muscles from 6 to 8-week-old wild-type and *Zeb1* (+/-) mice were dissected, pooled together and digested with Collagenase Type I (Sigma-Aldrich) and Dispase II (Sigma-Aldrich). The resulting cell suspension was depleted of erythrocytes using Red Blood Lysis Buffer © (Sigma-Aldrich) and processed for negative and positive selection of satellite cells in a FACS Aria™ II cell sorter (BD Biosciences). The antibodies used in MuSC isolation (see Supplementary Table 1 and figure legends) were used at the following dilutions/concentration from the commercially available product: α7 integrin (1/500), CD11b-PE (1/1000), CD31-PE (1/1000), CD34-biotin (1/250), CD45-PE (1/100), Sca1-PE (1/1000), Streptavidin-APC-Cy7 (1/200), and anti-mouse Alexa Fluor 647 (1/250). Sorted MuSC were cultured as described (21). Briefly, MuSC were plated on gelatin-coated dishes in a medium—commonly referred as standard growth medium for satellite cells (SGMSC)—that allows both proliferation and differentiation and that contains DMEM, 20% FBS (Sigma-Aldrich), 10% horse serum (Sigma-Aldrich), 1% penicillin/streptomycin, and 1% chicken embryo extract (SeraLab, West Sussex, UK). To assess their proliferation, MuSCs were labeled for 1 h with 1.5μg/mL of BrdU (Sigma-Aldrich) and pelleted. Two μm sections were prepared and assessed for BrdU incorporation as the percentage of $BrdU^+$ cells out of the total number of cells. To induce *ex vivo* differentiation of MuSCs, isolated MuSCs were plated on 48-well plates coated with gelatin (Sigma-Aldrich) and grown in F-10 Ham's medium (Lonza) supplemented with 20% FBS (Sigma-Aldrich), 1% Pen/Strep (Lonza), and 2.5 ng/ml basic FGF (Miltenyi Biotec, Bergisch Gladbach, Germany). Once MuSCs reached confluence, the above medium was replaced by DMEM supplemented with 2% of horse serum (Sigma-Aldrich) and 1% Pen/Strep and cells were cultured for the indicated period before they were assessed for gene expression by qRT-PCR. In selected experiments the effect of conditioned medium from macrophages on MuSC gene expression was examined as follows (see scheme in Fig. 8B). Macrophages infiltrating the gastrocnemius of wild-type and *Zeb1* (+/-) mice were sorted as described above and cultured during 24 h in SGMSC described above. Equal volumes of the conditioned medium produced by these macrophages was then added to wild-type MuSCs plated on gelatin-coated 48 well plates for 24 h before the latter were assessed for gene expression by qRT-PCR.

Transplant of MuSCs

Wild-type and *Zeb1* (+/-) MuSCs were seeded immediately after FACS isolation onto 24 well-plates in SGMSC medium and infected with lentivirus for CMV-GFP (a kind gift from J. Blanco (IQAC-CSIC, Barcelona, Spain) in the presence of 2.5μg/mL polybrene (Sigma-Aldrich). After 16 h the medium was replaced with fresh SGMSC medium and 1.5×10^4 cells were transplanted into the gastrocnemius of mdx recipient mice that had been injected with CTX the day before as previously described (6). Four weeks later, mice were sacrificed and the gastrocnemius muscle was dissected for further analysis.

Supplementary Table 1

Primary and secondary antibodies

Antibodies		
**Primary Abs**	**Source**	**Clone (Catalog Number)**
α7 integrin	MBL International	3C12 (K0046-3)
α-tubulin	Sigma-Aldrich	T6074
BrdU-FITC	BD Biosciences	B44 (347583)
CD3-APC	Tonbo Biosciences	145-2C11
CD4	Santa Cruz Biotechnologies	GK1.5 (sc-13573)
CD11b-PE	ImmunoTools	M1/70.15 (22159114)
CD31-PE	BD Biosciences	MEC 13.3 (553373)
CD34-Biotin	eBioscience	RAM34 (13-0341-82)
CD45	Santa Cruz Biotechnologies	2D-1 (sc-1187)
CD45-PE	eBioscience	RA3-6B2 (12-0452-81)
CD45-PerCP Cy5.5	Biolegend	30-F11
CD170 (SIGLECF)-PerCP eFluor 710	eBioscience	1RNM44N (46-1702-80)
CD206 (MRC1)-Alexa 488	AbD Serotec	MR5D3
Dystrophin	Santa Cruz Biotechnologies	MANDRA-1 (sc-73592)
F4/80	eBioscience	BM8 (14-4801-82)
F4/80-APC	BioLegend	BM8 (123116)
FOXP3	Santa Cruz Biotechnologies	2A11G9 (sc-53876)
GAPDH	Cell Signaling	14C10 (2118)
GFP	Aves Laboratories	GFP (GFP-1020)
Laminin	Santa Cruz Biotechnologies	4H8-2 (sc-59854)
Ly6C-PerCP-Cy5.5	eBioscence	HK1.4 (45-5932-82)
Ly6G-FITC	BC Biosciences	1A8
MHC (eMHC)	Developmental Studies Hybridoma Bank	F1.652
MYOD1	Santa Cruz Biotechnologies	C-20 (sc-304)
MYOG	Santa Cruz Biotechnologies	G-20 (sc-31945)
NCAM	R&D Systems	AF2408
NK1.1-FITC	BD Biosciences	PK136
p38	Sigma-Aldrich	M0800
Pax7	Developmental Studies Hybridoma Bank	Pax7
Phospho p38	Cell Signaling	9211L
Sca1-PE	BD Biosciences	D7 (553108)
ZEB1	Santa Cruz Biotechnologies	H-102 (sc-25388)
ZEB1	Santa Cruz Biotechnologies	E-20 (sc-10572)
ZEB1	Sigma-Aldrich	HPA027524
**Control Abs**	**Source**	**Catalog Number**
IgG mouse	Jackson ImmunoResearch	015-000-002
**Secondary Abs**	**Source**	**Catalog Number**
Anti-chicken Alexa Fluor 488	Jackson ImmunoResearch	103-545-155
Anti-goat TRITC	Jackson ImmunoResearch	705-025-147
Anti-mouse Alexa Fluor 488	Jackson ImmunoResearch	715-545-150
Anti-mouse Alexa Fluor 647	Thermo Fisher Scientific	A-21240
Anti-mouse HRP	Jackson ImmunoResearch	715-035-151
Anti-mouse Rhodamine Red-X	Jackson ImmunoResearch	715-295-151
Anti-mouse Dylight 649	Jackson ImmunoResearch	715-495-151
Anti-rabbit Alexa Fluor 488	Jackson ImmunoResearch	711-545-152
Anti-rabbit HRP	Jackson ImmunoResearch	111-035-144
Anti-rat Dylight 649	Jackson ImmunoResearch	112-495-175
Anti-rat Rhodamine Red-X	Jackson ImmunoResearch	712-295-153
Streptavidin APC Cy7	BD Biosciences	554063

Supplementary Table 2

DNA primers used in qRT-PCR

Gene	Forward 5' – 3'	Reverse 5' – 3'	Reference
Ccl2	GGGATCATCTTGCTGGTGAA	AGGTCCCTGTCATGCTTCTG	9
Cdh1	AGACTTTGGTGTGGGTCAGG	ATCTGTGGCGATGATGAGAG	8
Cdh2	AGCGCAGTCTTACCGAAGG	TCGCTGCTTTCATACTGAACTTT	12
Cdh15	ATGTGCCACAGCCACATCG	TCCATACATGTCCGCCAGC	14
Cdkn1c	ACCCCGCGCAAACGT	AGATGCCCAGCAAGTTCTCTCT	26
Cx3cr1	CTGCTCAGGACCTCACCAT	TTGTGGAGGCCCTCATGGCTGAT	11
Dusp1	TGGAGATCCTGTCCTTCCTG	AAGCTGAAGTTCGGGGAGAT	22
Foxo3	GATAAGGGCGACAGCAACAG	CTGTGCAGGGACAGGTTGT	19
Gapdh	CGACTTCAACAGCAACTCCCACTCTTCC	TGGGTGGTCCAGGGTTTCTTACTCCTT	3
Hes1	GCGAAGGGCAAGAATAAATG	TGTCTGCCTTCTCTAGCTTGG	16
Hes6	GCCGGATTTGGTGTCTACAT	TCCTGAGCTGTCTCCACCTT	20
Ifng	ATGAACGCTACACACTGCATC	CCATCCTTTTGCCAGTTCCTC	28
Il6	AACGATGATGCACTTGCAGA	TGGTACTCCAGAAGACCAGAGG	17
Il10	ACCAGCTGGACAACATACTGC	TCACTCTTCACCTGCTCCACT	4
Mrc1	CCATTTATCATTCCCTCAGCAAGC	AAATGTCACTGGGGTTCCATCACT	13
Myh3	CTTCACCTCTAGCCGGATGGT	AATTGTCAGGAGCCACGAAAAT	30
Myh4	ACAGACTAAAGTGAAAGCC	CTCTCAACAGAAAGATGGAT	1
Myod1	TGGGATATGGAGCTTCTATCGC	GGTGAGTCGAAACACGGGTCAT	10
Nos2	GACGAGACGGATAGGCAGAG	GCACATGCAAGGAAGGGAAC	2
Pax7	GAGAAACCCCGGGATGTTCAG	ATCCAGACGGTTCCCTTTCT	24
Tnf	AGGGTCTGGGCCATAGAACT	CCACCACGCTCTTCTGTCTAC	7
Zeb1	AACTGCTGGCAAGACAAC	TTGCTGCAGAAATTCTTCCA	24

Supplementary Table 3

Oligonucleotides for ChIP assays

Promoter Region	Forward 5' → 3'	Reverse 5' → 3'
Ccl2 promoter<br>*ZEB1 binding site (-1526bp)*<br>*(-1600/-1479 bp)*	CTCGAAAAACCAAAAAACAAATCT	GGCATAGTCAGTTGTCAA
Foxo3 promoter<br>*ZEB1 binding site (-404 bp)*<br>*(-437/-369 bp)*	CGGCCAGTGTTTCCACGCCCAGGTAA	AGTATCGGGTTCCTTATTAGAACCTGACACC

Supplementary Table 4

siRNA oligonucleotides

Gene Target	Upstream sequence	Reference
siCtl	GGUUACGAACUAAGCUAUA	24
siZEB1-A	GACCAGAACAGUGUUCCAUGUUUAA	24
siZEB1-B	AACUGAACCUGUGGAUUAU	24
siZEB1-C	CCUGUGGAUUAUGAGUUCA	24

REFERENCES FOR SUPPLEMENTARY METHODS

1. Abe, S. et al. Relationship between function of masticatory muscle in mouse and properties of muscle fibers. *Bull. Tokyo. Dent. Coll.* **49,** 53-58 (2008).

2. Aoshiba, K. & Nagai, A. Chronic lung inflammation in aging mice. *FEBS Lett.* **581,** 3512-3516 (2007).

3. Banerjee, S. et al. MicroRNA let-7c regulates macrophage polarization. *J. Immunol.* **190,** 6542-6549 (2013).

4. Bencze, M. et al. Proinflammatory macrophages enhance the regenerative capacity of human myoblasts by modifying their kinetics of proliferation and differentiation. *Mol. Ther.* **20,** 2168-2179 (2012).

5. Bulfield, G., Siller, W.G., Wight, P.A. & Moore, K.J. X chromosome-linked muscular dystrophy (mdx) in the mouse. *Proc. Nat. Acad. Sci. U.S.A.* **81,** 1189-92 (1984).

6. Cerletti, M. et al. Highly efficient, functional engraftment of skeletal muscle stem cells in dystrophic muscles. *Cell* **134,** 37-47 (2008).

7. Chen, H. et al. Anti-inflammatory effects of chicanine on murine macrophage by down-regulating LPS-induced inflammatory cytokines in IκBα/MAPK/ERK signaling pathways. *Eur. J. Pharmacol.* **724,** 168-174 (2014).

8. Cortés, M. et al. Tumor-associated macrophages (TAMs) depend on ZEB1 for their cancer-promoting roles. *EMBO J.* **36,** 3336-3355 (2017).

9. Deguchi, A. et al. Serum amyloid A3 binds MD-2 to activate p38 and NF-κB pathways in a MyD88-dependent manner. *J. Immunol.* **191,** 1856-1864 (2013).

10. Dogra, C., Changotra, H., Mohan, S. & Kumar, A. Tumor necrosis factor-like weak inducer of apoptosis inhibits skeletal myogenesis through sustained activation of nuclear factor-κB and degradation of MyoD protein. *J. Biol. Chem.* **281,** 10327-10336 (2006).

11. Fang, I.M., Lin, C.P., Yang, C.M., Chen, M.S. & Yang, C.H. Expression of CX3C chemokine, fractalkine, and its receptor CX3CR1 in experimental autoimmune anterior uveitis. *Mol. Vis.* **11,** e51 (2005).

12. Haug, J. S. et al. N-cadherin expression level distinguishes reserved versus primed states of hematopoietic stem cells. *Cell stem cell* **2,** 367-379 (2008).

13. Hayes, E.M. et al. Classical and alternative activation and metalloproteinase expression occurs in foam cell macrophages in male and female ApoE null mice in the absence of T and B lymphocytes. *Front. Immunol.* **5,** 147-152 (2015).

14. Hill, M., & Goldspink, G. Expression and splicing of the insulin-like growth factor gene in rodent muscle is associated with muscle satellite (stem) cell activation following local tissue damage. *J. Physiol.* **549,** 409-418 (2003).

15. Matsuda, R., Nishikawa, A. & Tanaka, H. Visualization of dystrophic muscle fibers in mdx mouse by vital staining with Evans blue: evidence of apoptosis in dystrophin-deficient muscle. *J. Biochem.* **118,** 959-64 (1995).

16. Murta, D. et al. Dynamics of Notch signalling in the mouse oviduct and uterus during the oestrous cycle. *Reprod. Fertil. Dev.* **28,** 1663-1678 (2016).

17. Nakajima, A. et al. Induction of interferon-induced transmembrane protein 3 gene expression by lipopolysaccharide in astrocytes. *Eur. J. Pharmacol.* **745,** 166-175 (2014).

18. Novak, M. L., Weinheimer-Haus, E. M., & Koh, T. J. Macrophage activation and skeletal muscle healing following traumatic injury. *J. Pathol.* **232**, 344-355 (2014).

19. Nowak, K., Killmer, K., Gessner, C., & Lutz, W. E2F-1 regulates expression of FOXO1 and FOXO3a. *Biochimica et Biophysica Acta (BBA)-Gene Structure and Expression.* **1769,** 244-252 (2007).

20. Ogura, Y. et al. TAK1 modulates satellite stem cell homeostasis and skeletal muscle repair. *Nat. Commun.* **6,** 10123 (2015).

21. Pasut, A., Jones, A. E., & Rudnicki, M. A. Isolation and culture of individual myofibers and their satellite cells from adult skeletal muscle. *J. Vis. Exp.* e50074 (2013).

22. Perdiguero, E. et al. p38/MKP-1–regulated AKT coordinates macrophage transitions and resolution of inflammation during tissue repair. *J. Cell. Biol.* **195,** 307-22 (2011).

23. Sánchez-Tilló, E. et al. β-catenin/TCF4 complex induces the epithelial-to-mesenchymal transition (EMT)-activator ZEB1 to regulate tumor invasiveness. *Proc. Natl. Acad. Sci. U.S.A.* **108,** 19204-19209 (2011).

24. Siles, L. et al. ZEB1 imposes a temporary stage-dependent inhibition of muscle gene expression and differentiation via CtBP-mediated transcriptional repression. *Mol. Cell. Biol.* **33,** 1368-1382 (2013).

25. Takagi, T., Moribe, H., Kondoh, H. & Higashi, Y. δEF1, a zinc finger and homeodomain transcription factor, is required for skeleton patterning in multiple lineages. *Development* **125,** 21-31 (1998).

26. Tury, A., Mairet-Coello, G. & DiCicco-Bloom, E. The cyclin-dependent kinase inhibitor p57Kip2 regulates cell cycle exit, differentiation, and migration of embryonic cerebral cortical precursors. *Cereb. Cortex*. **21,** 1840-1856 (2011).

27. Villalta, S. A., Rinaldi, C., Deng, B., Liu, G., Fedor, B., & Tidball, J. G. Interleukin-10 reduces the pathology of mdx muscular dystrophy by deactivating M1 macrophages and modulating macrophage phenotype. *Hum. Mol. Genet.*, **20**, 790-805 (2011).

28. Ydens E, Cauwels A, Asselbergh B, Goethals S, Peeraer L, Lornet G, Almeida-Souza L, Van Ginderachter JA, Timmerman V, Janssens S.. Acute injury in the peripheral nervous system triggers an alternative macrophage response. *J. Neuroinflammation* **9,** 176 (2012).

29. Zhang, X., Goncalves, R. & Mosser, D.M. The isolation and characterization of murine macrophages. *Curr. Protoc. Immunol.* 14-1 (2008).

30. Zhou, Y. et al. An altered phenotype in a conditional knockout of Pitx2 in extraocular muscle. *Invest. Ophthalmol. Vis. Sci*. **50,** 4531-4541 (2009).

SUPPLEMENTARY FIGURES

Supplementary Figure 1A-E

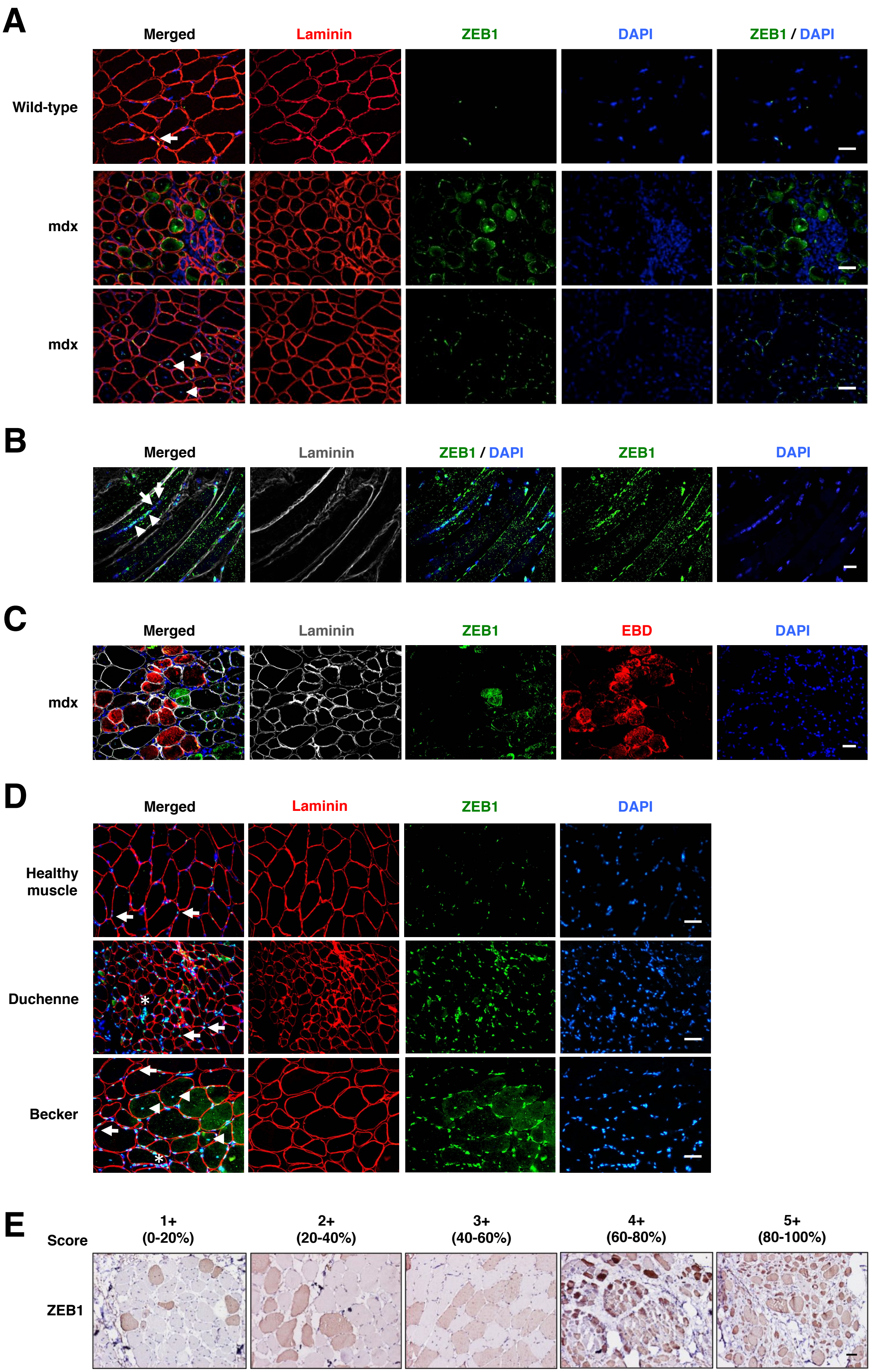

Supplementary Figure 1F-J

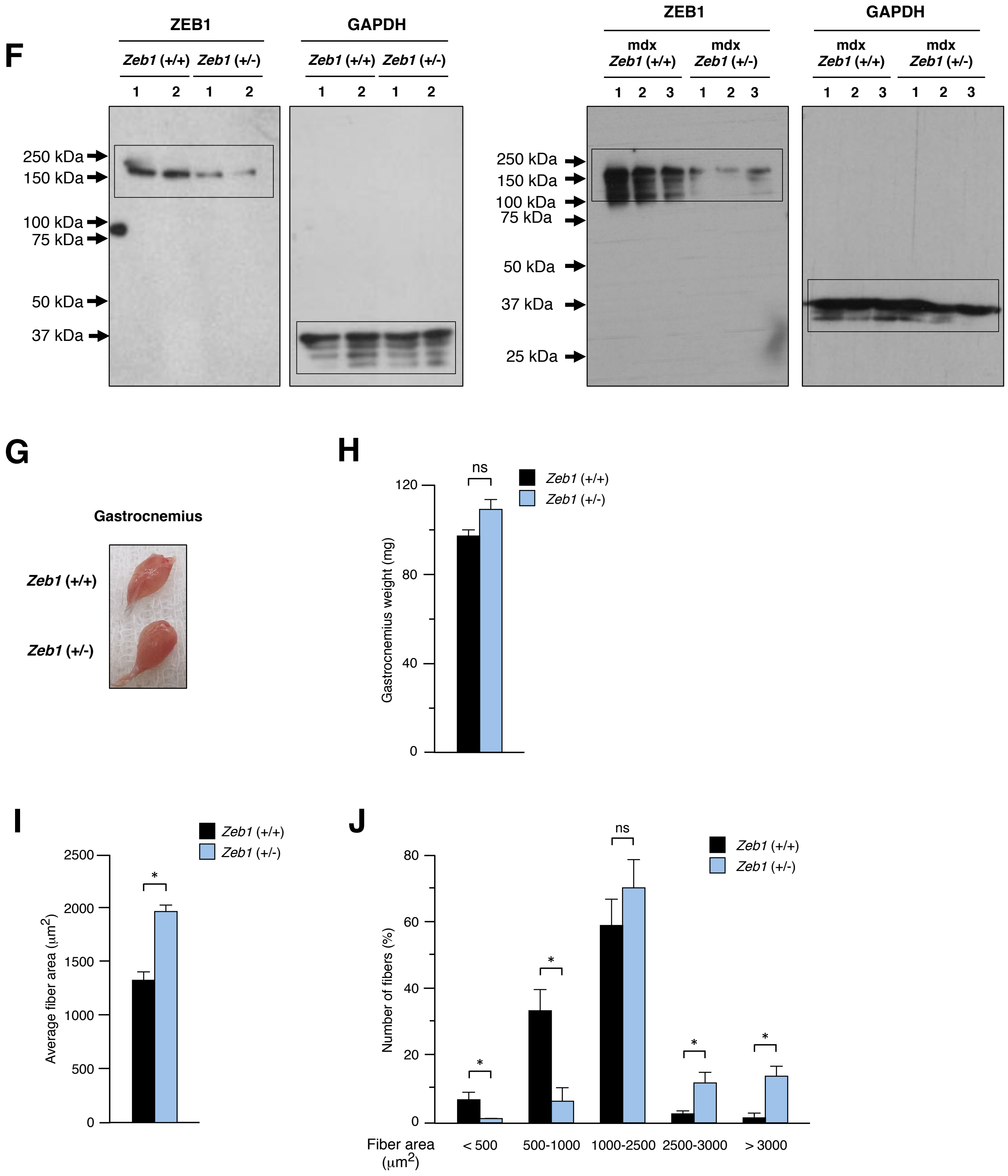

SUPPLEMENTARY FIGURE 1

ZEB1 is upregulated in dystrophic muscles and is expressed by undamaged and regenerating myofibers. (A) Individual staining for laminin, ZEB1 and/or DAPI as well as for combined ZEB1/DAPI corresponding to Fig. 1B. Scale bars: 25 μm (wild-type mice) 50 μm (mdx mice). **(B)** Myofibers can harbor $ZEB1^{+}$ and $ZEB1^{-}$ nuclei. Staining for ZEB1 (clone H-102), laminin (clone 4H8-2) and DAPI in a longitudinal section of the gastrocnemius of 2-month-old mdx mice. Staining for laminin (clone 4H8-2) was followed by an anti-rat Dylight 649 that was then converted to grey for representation purposes. Pictures are representative of at least three mice. Scale bar: 25 μm. **(C)** Single staining captures for Fig. 1D. As in (B), staining for laminin was converted to grey for representation purposes. Scale bar: 50 μm. **(D)** Single staining captures for Fig. 1E. Scale bars: 50 μm. **(E)** The relative number of myofibers (as a percentage, 0% to 100%) expressing ZEB1 in human muscular dystrophy biopsies was coded into a score (1+ to 5+) and used in Fig. 1F and 1G. Human muscular dystrophy biopsies were stained for ZEB1 (clone H102) followed by and anti-rabbit HRP-conjugated secondary antibody. The pictures shown are representative from biopsies with different levels of ZEB1. Scale bar: 50 μm. **(F)** Full unedited blots for Fig. 1H. **(G)** The gastrocnemius muscles of 2-months-old wild-type and *Zeb1* (+/-) mice displayed similar overall macroscopic structure. The picture shown is representative of at least 6 mice for each genotype. **(H)** As in (G), but the gastrocnemius muscles of mice from both genotypes were assessed for their weight. Data shown are representative of at least 6 mice for each genotype. **(I)** *Zeb1* (+/-) myofibers have a larger average size than wild-type ones. The gastrocnemius muscles of four mice for each genotype were analyzed for their cross-sectional area (CSA) as described in Supplementary Methods. **(J)** Myofibers in *Zeb1* (+/-) gastrocnemius muscles have a higher share of larger size myofibers and a lower share of smaller size myofibers. Data shown are representative of four mice for each genotype.

Supplementary Figure 2A-H

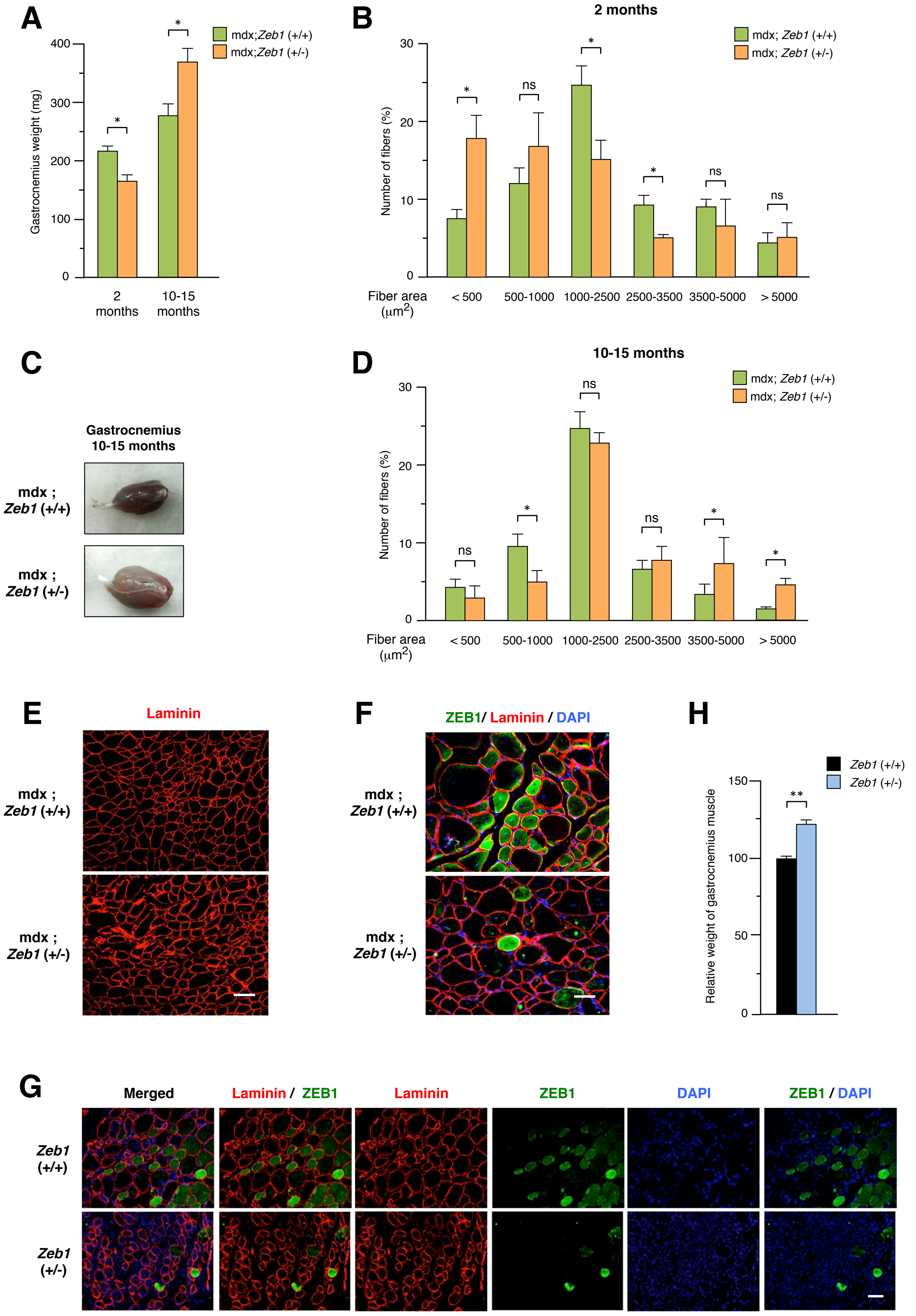

SUPPLEMENTARY FIGURE 2

ZEB1 protects dystrophic and acutely injured muscles from damage. (A) At 2 months of age, the gastrocnemius of mdx;*Zeb1* (+/-) mice were lighter than those of mdx;*Zeb1* (+/+) counterparts, the reverse at 10-15 months of age. Data are the mean of six mice for each genotype. **(B)** The gastrocnemius muscles of 2-month-old mdx;*Zeb1* (+/-) mice have a larger share of smaller size myofibers than mdx;*Zeb1* (+/+) mice. CSA and myofiber size distribution were conducted as indicated in Supplementary Methods. Data are the mean of at least five mice for each genotype. **(C)** The gastrocnemius muscles of 10-15-month-old mdx;*Zeb1* (+/-) and mdx;*Zeb1* (+/+) mice are shown. Pictures are representative of at least six mice for each genotype. The muscles shown had been injected with EBD 9 to12 h before euthanasia. **(D)** The gastrocnemius muscles of 10-15-month-old mdx;*Zeb1* (+/-) have a greater share of larger size myofibers than those of mdx;*Zeb1* (+/+) mice of the same age. Data are the mean of at least five mice for each genotype. (**E)** Muscles from 2-month-old mdx;*Zeb1* (+/+) and mdx;*Zeb1* (+/-) mice both contain myofibers of different sizes but myofiber abnormalities were more evident in the latter. Samples were stained for laminin (clone 4H8-2). Scale bar: 100 μm. **(F)** Expression of ZEB1 was lower in mdx;*Zeb1* (+/-) muscles than in mdx;*Zeb1* (+/+) counterparts. The gastrocnemius muscles of 2-month-old mice from both genotypes were stained for ZEB1 (clone H-102), laminin (clone 4H8-2) and DAPI. Scale bar: 50 μm. **(G)** Individual staining captures for ZEB1, laminin and/or DAPI corresponding to Fig. 2G. Scale bar: 50 μm. **(H)** Weight of the gastrocnemius muscles of 2-month-old wild-type and *Zeb1* (+/-) mice treated with CTX during 48 h. Data are the mean of least six mice for each genotype.

Supplementary Figure 3A-C

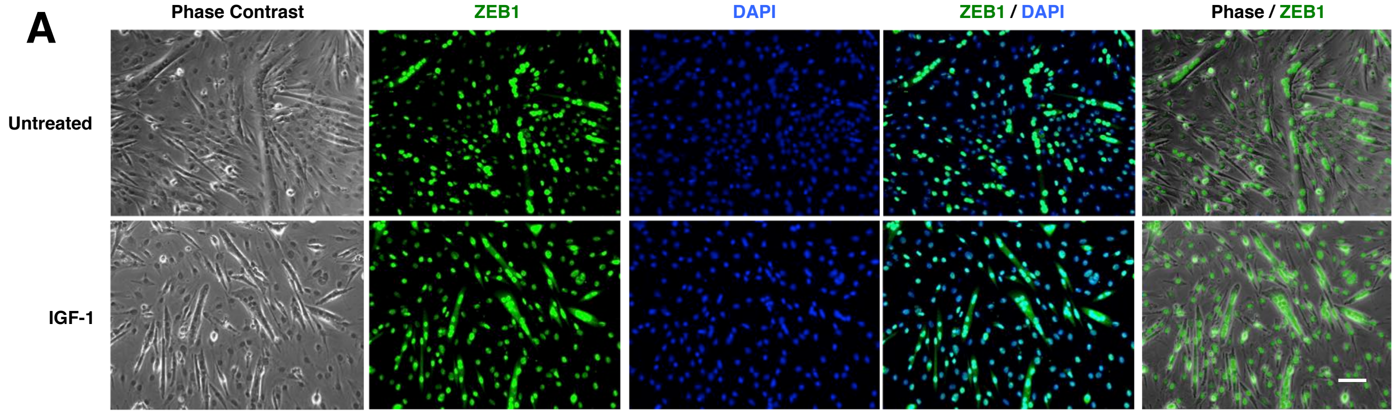

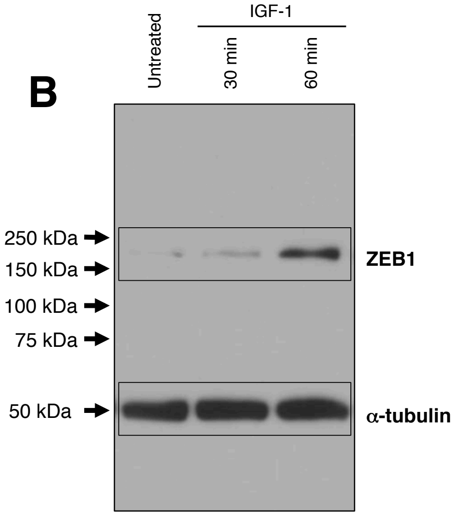

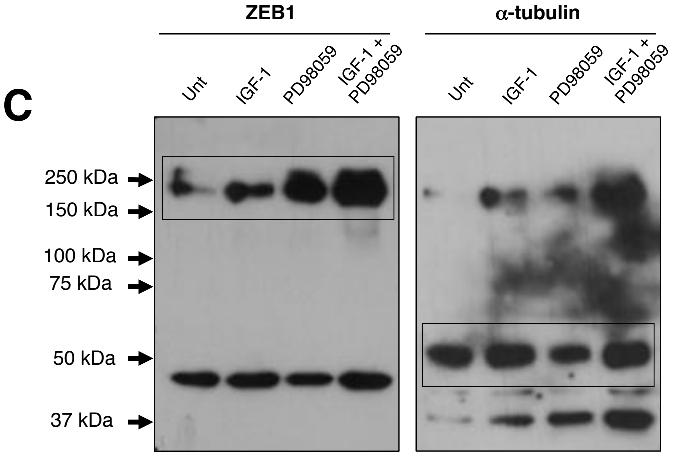

SUPPLEMENTARY FIGURE 3

IGF-1 upregulates ZEB1 expression and promotes its cytoplasmic translocation in an ERK/MEK-dependent manner. (A) Individual phase contrast and immunofluorescence staining captures for ZEB1 (H-102) and DAPI corresponding to Fig. 3A. Scale bar: 50 μm. **(B)** Full unedited blot for the *right panel* of Fig. 3B. The membrane shown was subsequently blotted first for ZEB1 (HPA027524) and, without previous stripping, then for α-tubulin (T6074). **(C)** Full unedited blots for Fig. 3D.

Supplementary Figure 4A

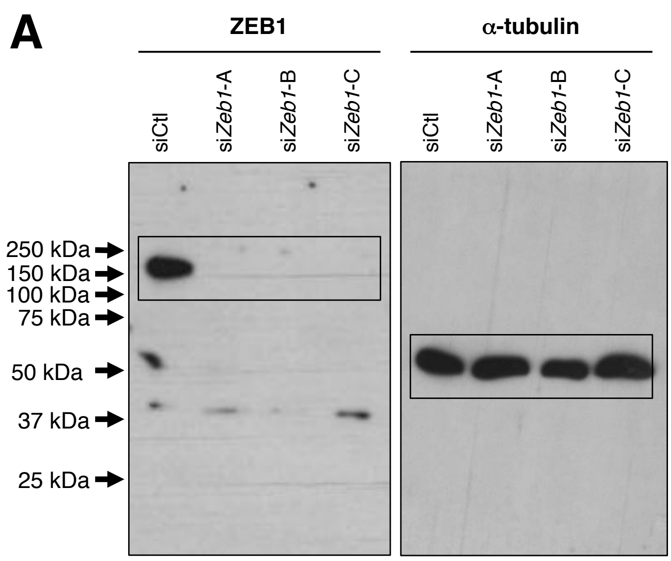

SUPPLEMENTARY FIGURE 4

Knockdown efficiency for siRNAs in Fig. 4G. (A) C2C12 cells were transfected with siCtrl or three specific siRNAs against ZEB1 (24). Cell lysates were then blotted for ZEB1 (clone HPA027524) along with α-tubulin (clone T6074) as loading control.

Supplementary Figure 5A-I

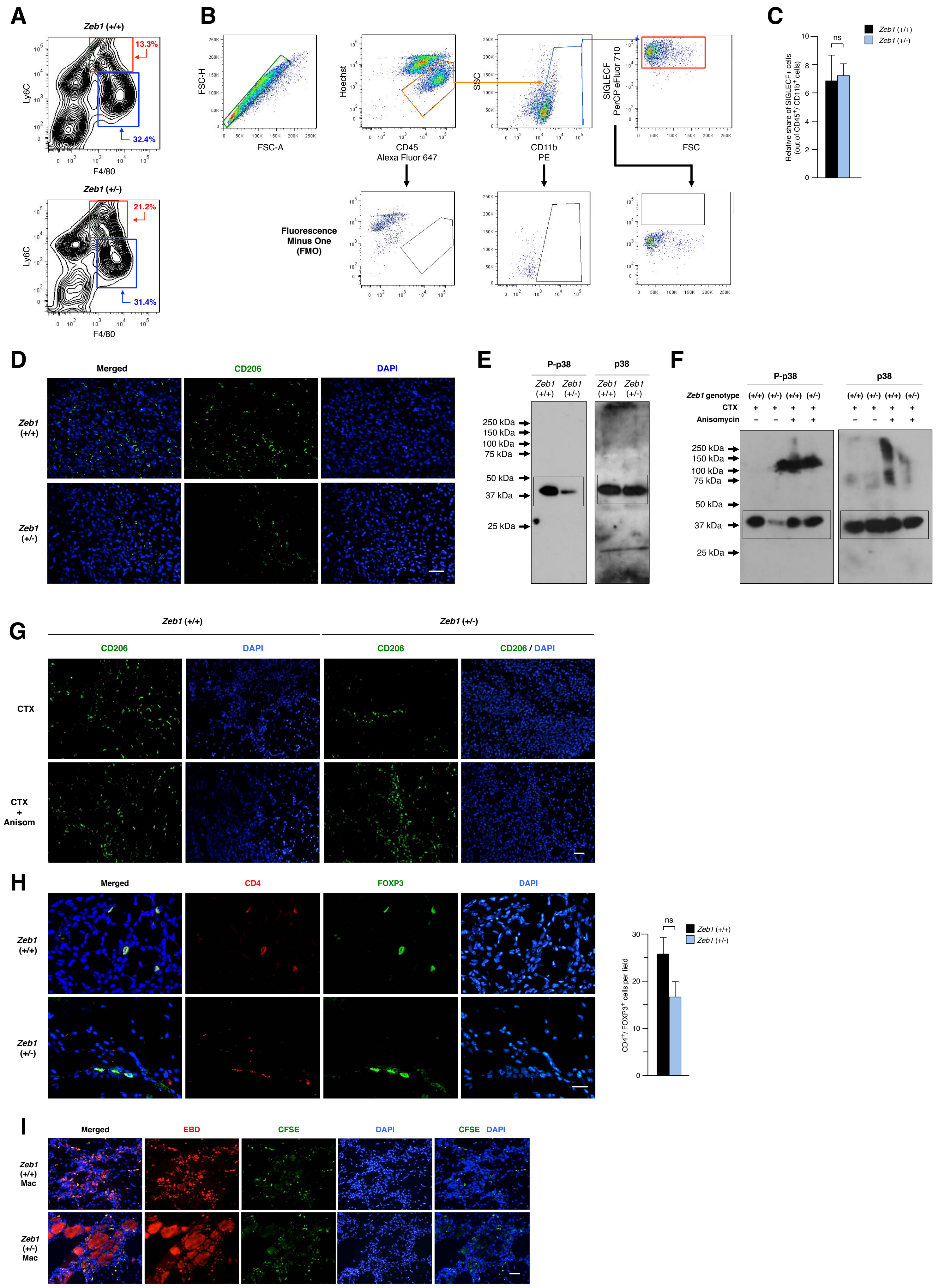

SUPPLEMENTARY FIGURE 5

ZEB1 expression in infiltrating macrophages accelerates their p38-dependent transition toward an anti-inflammatory phenotype and reduces muscle damage upon their transplant. (A) As in Fig. 5D but for all F4/80 and Ly6C subpopulations. **(B)** Wild-type and *Zeb1* (+/-) injured muscles contain a similar share of eosinophils. The eosinophil population in muscles from both genotypes two days post-CTX injection was characterized by CD170 (SIGLECF) staining as detailed in Supplementary Methods. *Upper panel*: Representative plots of the FACS staining strategy are shown. *Lower panel*: Fluorescence Minus One controls. **(C)** As in (B), quantification of eosinophils (SIGLECF$^+$ cells) out of all CD45$^+$ CD11b$^+$ cells. Data shown are the average of three mice for each genotype. **(D)** As in Fig. 5E, representative immunofluorescence captures of wild-type and *Zeb1* (+/-) injured muscles 7 days after CTX injection. Muscle sections were stained for CD206 (clone MR5D3) along with DAPI. Scale bar: 50 μm. **(E)** Full unedited blots for the *left panel* of Fig. 5F. **(F)** Full unedited blots for Fig. 5G. **(G)** Single staining captures for Fig. 5J**. (H)** Wild-type and *Zeb1* (+/-) muscles have a similar share of TReg (CD4$^+$ FOXP3$^+$) cells. *Left panel*: The gastrocnemius of wild-type and *Zeb1* (+/-) mice were stained for CD4 (clone GK1.5) and FOXP3 (clone 2A11G9) 48 h after CTX injection. Representative captures from at least three mice for each genotype are shown. *Right panel*: quantification of TReg cells from at least three mice for each genotype in the left panel. **(I)** Individual immunofluorescence staining captures for the *left panel* of Fig. 5K. Scale bar: 50 μm.

Supplementary Figure 6A-D

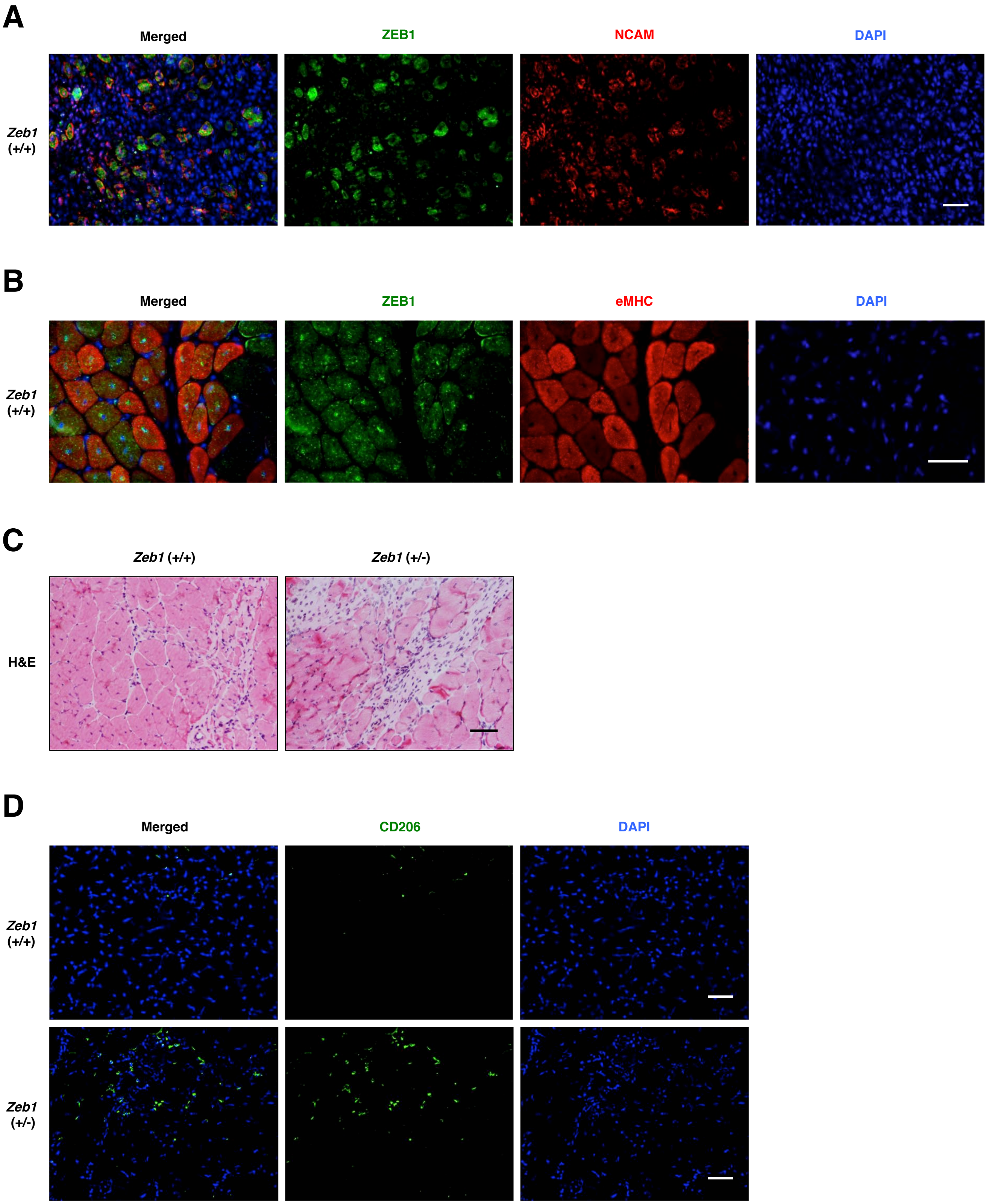

SUPPLEMENTARY FIGURE 6

ZEB1 is required for efficient muscle regeneration upon injury. (A) Single immunofluorescence staining captures for Fig. 6H. **(B).** Single immunofluorescence staining captures for Fig. 6I. **(C)** As in Fig. 6L. At day 14 after a second round of CTX-induced acute injury, and compared to wild-type counterparts, *Zeb1* (+/-) muscles displayed impaired regeneration as well as a more intense and extensive inflammatory infiltrate. The gastrocnemius muscles of mice from both genotypes were stained for hematoxylin/eosin. Scale bar: 50 µm. **(D)** Single staining for Fig. 6M.

Supplementary Figure 7A-D

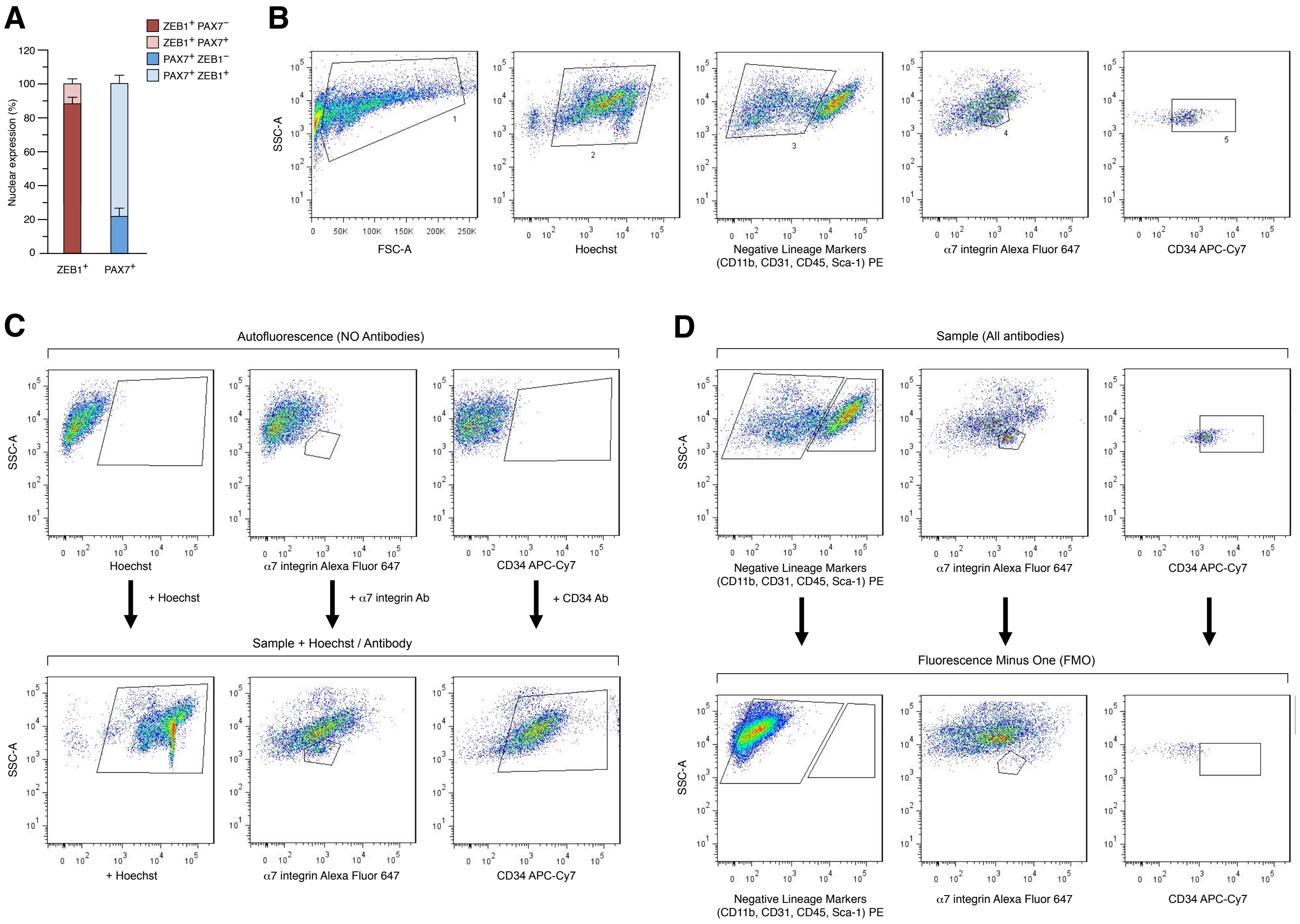

Supplementary Figure 7E-F

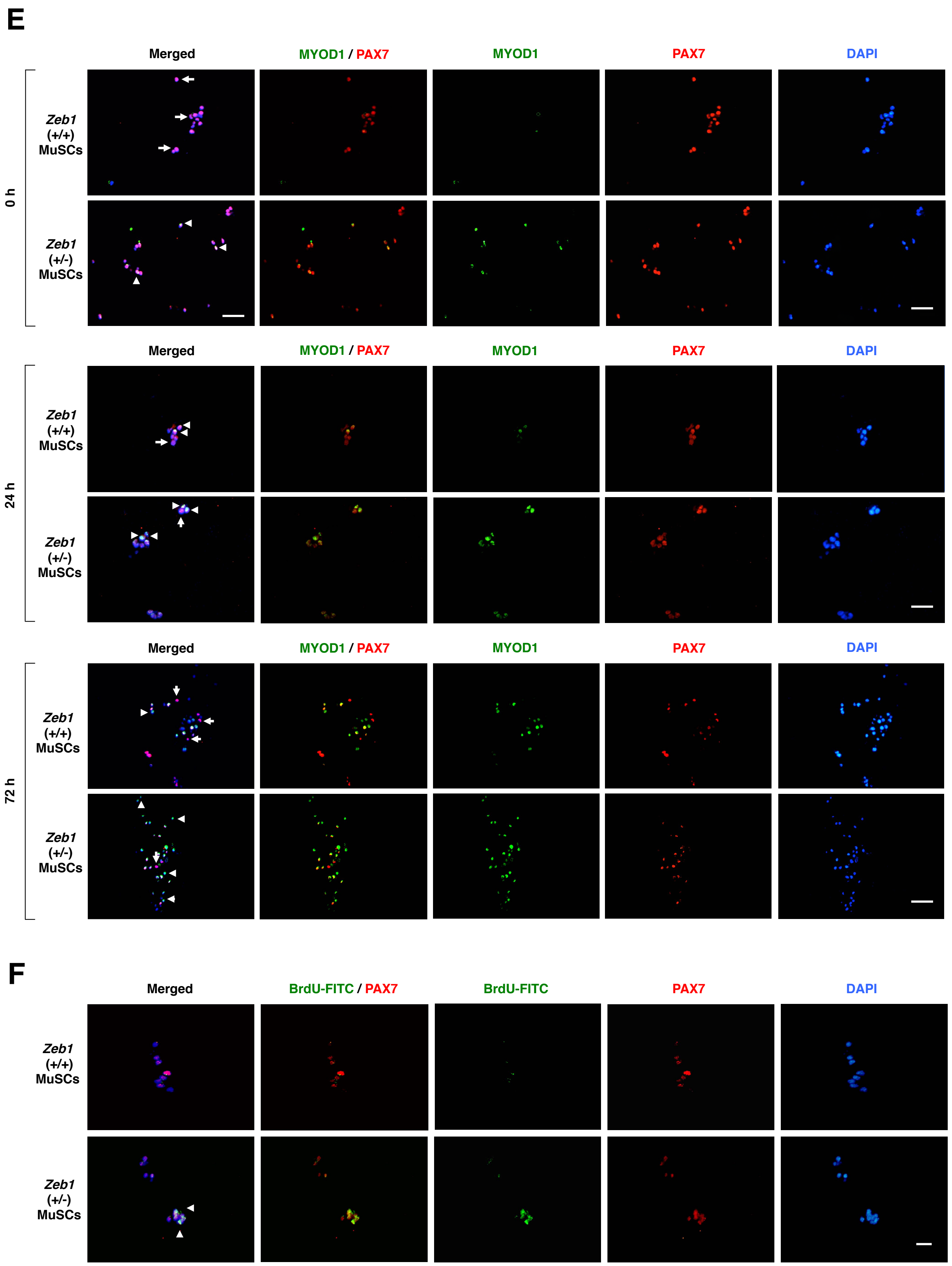

Supplementary Figure 7G-J

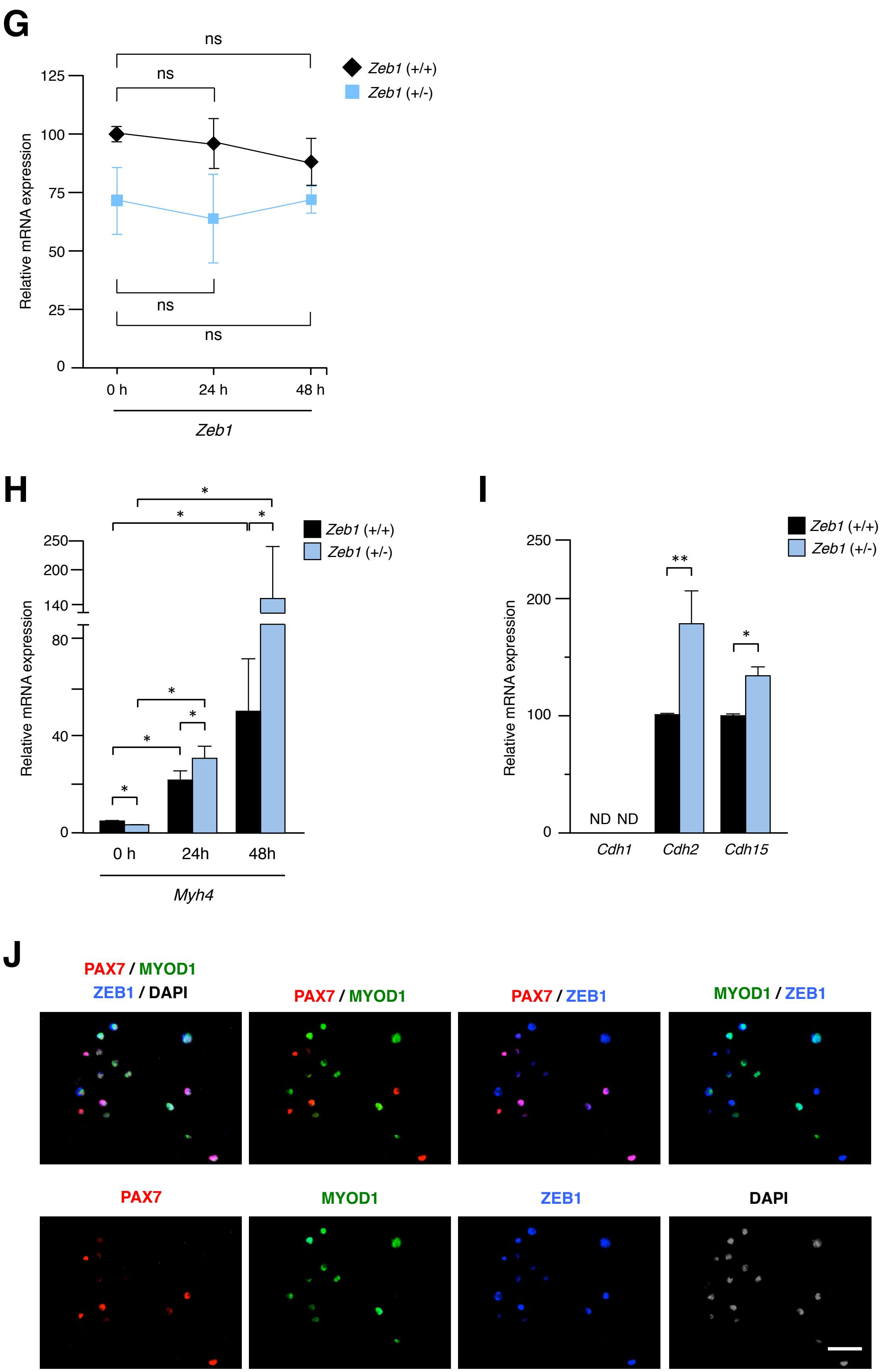

SUPPLEMENTARY FIGURE 7

Satellite cells require full levels of *Zeb1* to maintain their quiescence and to prevent their premature activation. (A) Expression of ZEB1 and PAX7 in the nuclei of wild-type muscles. The gastrocnemius muscles of 2-month-old wild-type mice were stained by immunofluorescence for ZEB1 (clone H-102), PAX7 (clone PAX7 DSHB), and DAPI. More than 400 nuclei from 6 mice were analyzed. At least five fields at 40X were quantified from four mice. **(B)** MuSCs were isolated by positive and negative selection as detailed elsewhere (21) and in Supplementary Methods. Representative plots of the sorting strategy are shown. **(C)** As in (B) but showing autofluorescence and single staining for Hoechst or each of the indicated antibodies. **(D)** As in (B) but showing fluorescence minus one (FMO) controls for each of the antibodies used. **(E)** Downregulation of *Zeb1* in MuSCs triggers their premature activation and myogenic progression. Representative immunofluorescence staining captures for Fig. 7D are shown. Individual representative cells of the different MuSC subpopulations were labeled with arrows ($PAX7^{+}$ $MYOD1^{-}$ cells) and arrowheads ($PAX7^{+}$ $MYOD1^{+}$ cells at 0 and 24 h and $PAX7^{-}$ $MYOD1^{+}$ cells at 72 h). Scale bar: 50 μm. **(F)** Downregulation of *Zeb1* in MuSCs increases BrdU uptake. Representative pictures for Fig. 7E are shown. Arrowheads mark $BrdU^{+}$ cells. Scale bar: 50 μm. **(G)** *Zeb1* expression remains stable during *in vitro* differentiation of MuSCs. Wild-type and *Zeb1* (+/-) MuSCs were cultured *ex vivo* until they reached confluence. At this point, their growth medium was replaced by differentiation medium (2% horse serum) for up to 48 h and *Zeb1* expression was assessed by qRT-PCR. Data are the average of MuSC cultures from 4 mice for each genotype and time. **(H)** *Myh4* is upregulated during *in vitro* differentiation of MuSCs but its levels are higher in *Zeb1* (+/-) MuSC cultures. As in (E), but for *Myh4*. **(I)** *Cdh2* and *Cdh15* are upregulated in *Zeb1* (+/-) MuSCs. Freshly isolated (0 h) wild-type and *Zeb1* (+/-) MuSCs were assessed for *Cdh1, Cdh2,* and *Cdh15* mRNA expression by qRT-PCR. mRNA levels in wild-type MuSCs were arbitrarily set to 100. ND: not detected. Data are the average of MuSC cultures from five mice for each genotype and time. **(J)** Individual and combination staining captures for Figure 7I. See main text and legend to Fig. 7I for details. Staining for ZEB1 (clone E-20), PAX7 (clone Pax7 DSHB), and MYOD1 (clone C20), was followed by their corresponding secondary antibodies conjugated to TRITC, DyLight 649, and Alexa Fluor 488, respectively. For representation purposes, DyLight 649 was converted into red, TRITC to blue, and nuclear staining with DAPI to grey. Scale bar: 50 μm.

Supplementary Figure 8A-B

A

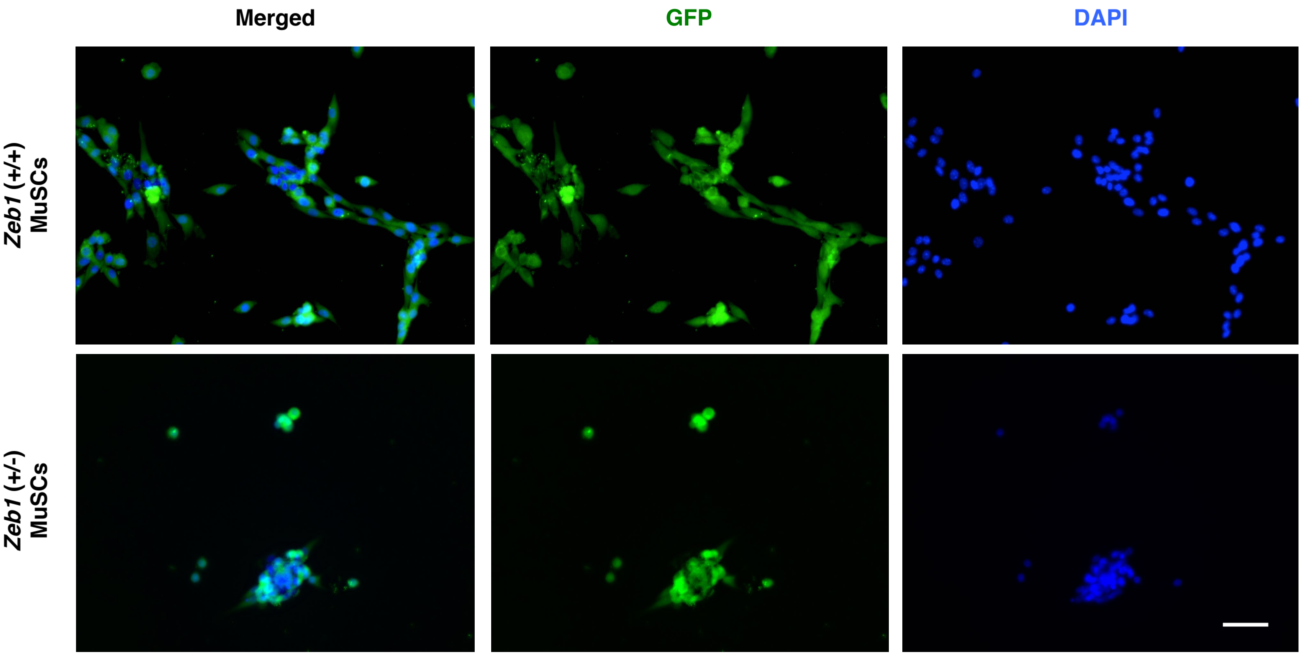

B

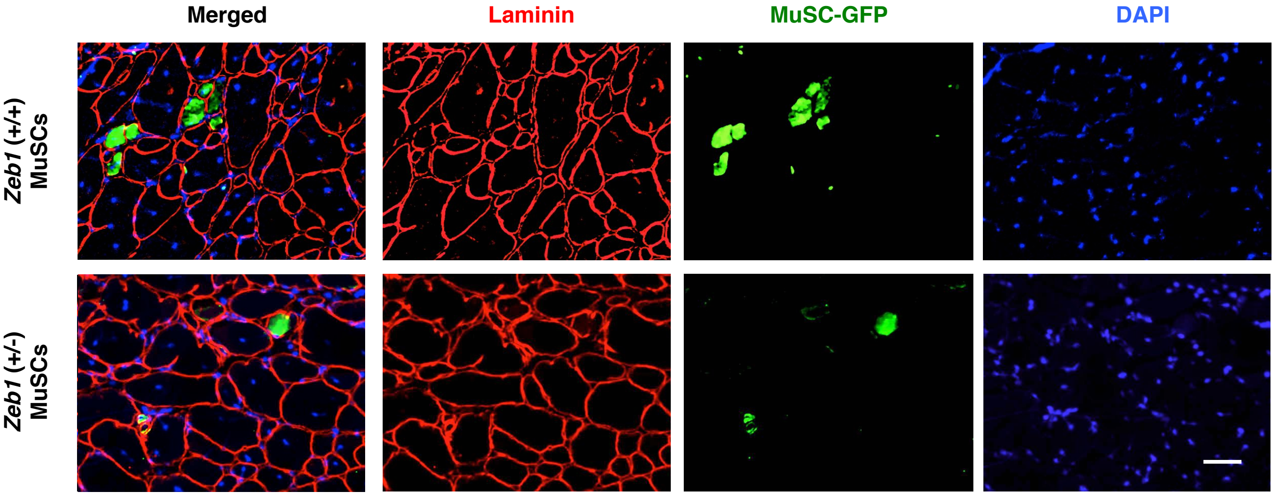

SUPPLEMENTARY FIGURE 8

MuSCs require full levels of *Zeb1* to drive efficient muscle regeneration. (A) GFP-labeled MuSCs from the gastrocnemius of wild-type and *Zeb1* (+/-) mice displayed similar levels of GFP expression. To enhance GFP fluorescence signal, cells were stained with an anti-GFP antibody (clone GFP-1020). Scale bar: 50 μm. **(B)** MuSCs depend on their expression of full levels of *Zeb1* to drive efficient muscle regeneration. Higher magnification of the merged and individual staining pictures corresponding to the inset in Fig. 8G. Muscle sections were stained for GFP (clone GFP-1020), laminin (clone 4H8-2) and/or DAPI. Scale bar: 50 μm.
